# Establishing quasi-steady state operations of microphysiological systems (MPS) using tissue-specific metabolic dependencies

**DOI:** 10.1038/s41598-018-25971-y

**Published:** 2018-05-22

**Authors:** Christian Maass, Matthew Dallas, Matthew E. LaBarge, Michael Shockley, Jorge Valdez, Emily Geishecker, Cynthia L. Stokes, Linda G. Griffith, Murat Cirit

**Affiliations:** 1 Department of Biological Engineering, Massachusetts Institute of Technology, Cambridge, MA USA; 2 Thermo Fisher Scientific, Frederick, MD USA; 3 Stokes Consulting, Redwood City, CA USA

## Abstract

Microphysiological systems (MPS), consisting of tissue constructs, biomaterials, and culture media, aim to recapitulate relevant organ functions *in vitro*. MPS components are housed in fluidic hardware with operational protocols, such as periodic complete media replacement. Such batch-like operations provide relevant nutrients and remove waste products but also reset cell-secreted mediators (e.g. cytokines, hormones) and potentially limit exposure to drugs (and metabolites). While each component plays an essential role for tissue functionality, MPS-specific nutrient needs are not yet well-characterized nor utilized to operate MPSs at more physiologically-relevant conditions. MPS-specific nutrient needs for gut (immortalized cancer cells), liver (human primary hepatocytes) and cardiac (iPSC-derived cardiomyocytes) MPSs were experimentally quantified. In a long-term study of the gut MPS (10 days), this knowledge was used to design operational protocols to maintain glucose and lactate at desired levels. This quasi-steady state operation was experimentally validated by monitoring glucose and lactate as well as MPS functionality. In a theoretical study, nutrient needs of an integrated multi-MPS platform (gut, liver, cardiac MPSs) were computationally simulated to identify long-term quasi-steady state operations. This integrative experimental and computational approach demonstrates the utilization of quantitative multi-scale characterization of MPSs and incorporating MPS-specific information to establish more physiologically-relevant experimental operations.

## Introduction

Recent advances in bioengineering, tissue engineering and microfabrication techniques have enabled the development of microphysiological systems (MPS), also known as tissue chips, organs-on-a-chip or body-on-a-chip. These *in vitro* systems aim to more comprehensively recapitulate essential physiological characteristics and functions of human tissues and organs. Current efforts have led to the development of numerous individual organ MPSs, including liver, kidney, skin and cardiac MPSs^1–10^ as well as several integrated multi-MPS platforms^5,11–19^. Potential applications of MPS technologies using healthy and diseased tissues include pharmacodynamics, safety pharmacology, and pharmacokinetics as well as basic research^14,20–28^.

In addition to the requisite cellular components, MPS technologies comprise several essential non-biological components to recreate the physiological microenvironment *in vitro*. These include i) hardware to house the tissue constructs and provide proper mechanical cues, ii) biomaterials to support functional tissue organization, and iii) cell culture medium to provide essential nutrients and facilitate cellular communication within and among MPSs.

The cell culture medium is among the most important support elements. The compositions of the most commonly used commercially available basal tissue culture media derive from the groundwork of Eagle^29^, who proposed, for the human carcinoma cell line HeLa, an optimal growth medium composition of various amino acids, salts, vitamins and other components. Since then, scores of basal media formulations have been developed to optimise culture parameters to support growth and other phenotypic behaviors of numerous cancer cell lines and primary epithelial and stromal cells used in basic research as well as CHO cells and hybridomas used in large scale production of proteins^30–37^.

The complex nature of MPSs, often involving multiple cell co-cultures and difficult to culture cell types (e.g., primary human and iPSC-derived) as well as the need for increased operational complexity (e.g. for interconnecting MPSs and running longer experiments), presents additional challenges for optimising MPS culture medium. MPS operational parameters for medium circulation and replacement are critically important as these parameters directly affect essential nutrient and waste concentrations. Complete media change intervals (i.e., medium is completely removed and replaced) range from 1 to 4 days for multi-MPS platforms^38–40^ and are typically 2 days for single MPSs^1,5,41^. While effective, complete media changes also remove cell-secreted mediators such as cytokines and hormones, important factors that modulate tissue behavior^5^. It may also be problematic for pharmacology studies since exogenous drugs and their metabolites are added and removed unrealistically. Further, lack of drug metabolite accumulation in the limited intervals between medium changes may result in incorrect assessment of metabolite-induced efficacy or toxicity, and consequently poor prediction of clinical effects.

An alternate medium change mode frequently used for microscale MPSs is continuous feed and removal of medium (flow-through without recirculation), with a very small volume resident in the MPS itself^2^. This provides fresh nutrients and removes wastes continuously, but also continuously removes cellular products and drug metabolites, again limiting MPS applications. Potentially, partial media change protocols, where a fraction of the MPS medium is replaced periodically, could still replenish nutrients and remove waste products sufficiently while better maintaining cell-secreted mediators, added drugs, and drug metabolites. In comparison to complete medium change protocols, this could reduce the required medium volume. Improving the cell-to-media ratio is desirable to provide a more physiologically-similar microenvironment. Partial media change protocols have been used with multi-MPS platforms^13,14,42^, however, no quantitative approach has been presented on how to rationally design such protocols to meet MPS specific nutrient or waste removal requirements. Given these challenges, the focus of this study is to investigate MPS-specific nutrient needs and develop operational strategies for maintaining limiting nutrients within desirable ranges in single MPSs and multi-MPS platforms.

In this study, MPS-specific nutrient utilization in three different established MPSs (liver, gut, cardiac^1,5,10^) was quantified while phenotypic metrics were monitored to assess tissue functionality and viability. Subsequently, mechanistic computational models were used to estimate rate constants for nutrient consumption and waste production. For the gut MPS, these models in combination with MPS-specific biological information were used to develop an alternative to the established two-day complete media change protocol (which is common practice for the investigated MPSs) to maintain limiting nutrients and waste products at desired quasi-steady state levels. The utility of the operational strategy was verified experimentally in terms of MPS functionality as well as intracellular metabolomics and proteomics in comparison to the common media change protocol. Lastly, a theoretical study was performed to define a new operational strategy to provide quasi-steady state operation in a theoretical 3-MPS platform with interconnected gut, liver, and cardiac MPSs.

Through this work, we have demonstrated that a combination of quantitative data and mechanistic computational models enables design of MPS operational protocols that should better maintain media components at desired quasi-steady state conditions and, potentially, enable longer term pharmacology studies. This approach is readily adaptable to regulate any media component and can be easily applied to any combination of interacting MPSs.

## Results

### Nutrient dependency assessment with individual gut, liver and cardiac MPSs

**Figure 1:**
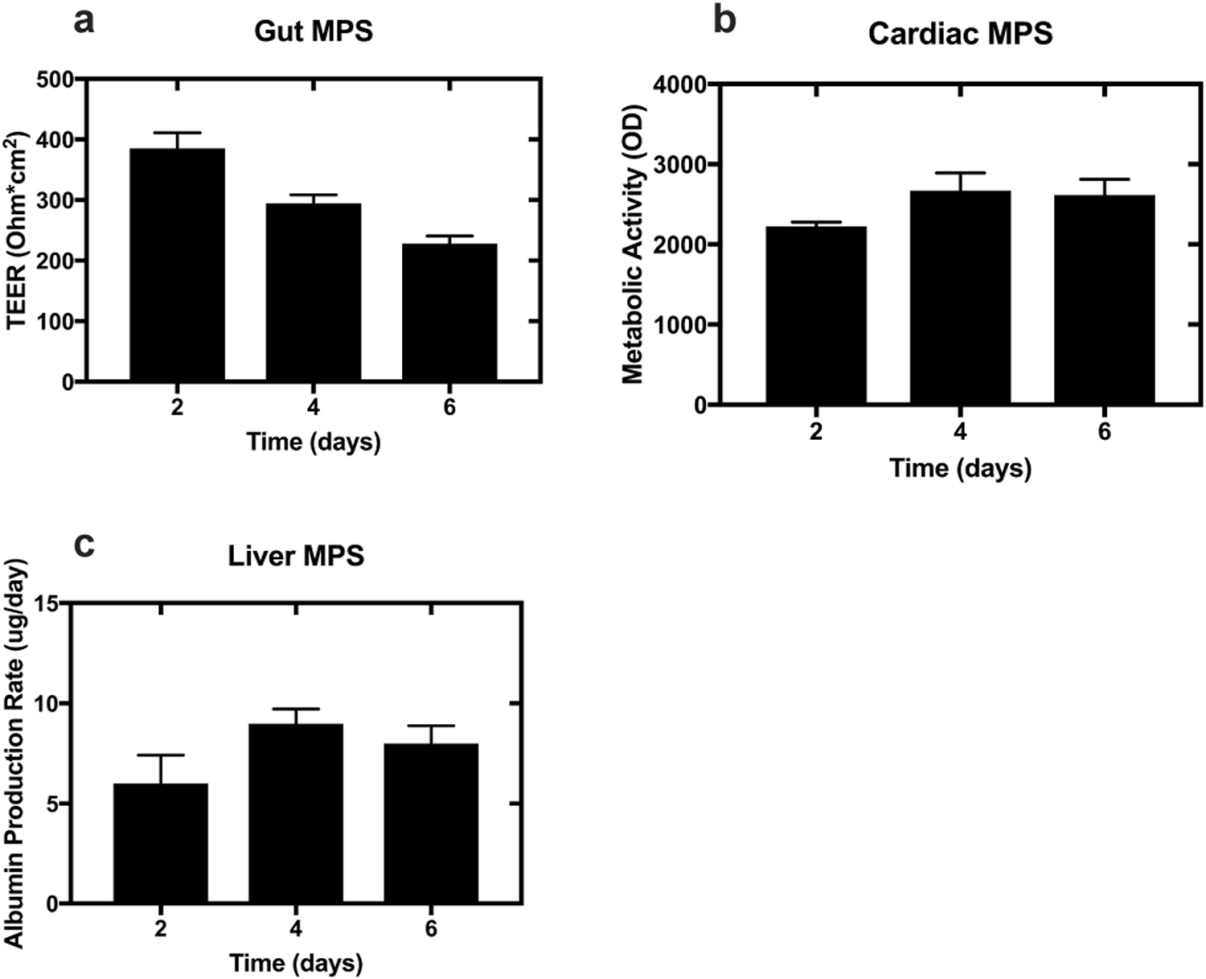
Assessment of MPS functionality without media change. The viability and functionality of gut MPS, cardiac MPS and liver MPS were assessed over six days without media change. (**a**) Gut MPS barrier integrity was monitored with TEER measurements. (**b**) Cardiac MPS viability was assessed with metabolic activity measurements using PrestoBlue. (**c**) Liver MPS functionality was assessed by albumin production rate. Error bars are standard deviation of triplicate MPSs for each time point.

In an extended experiment with no medium exchange, we investigated how tissue culture medium composition changed over time to identify limiting nutrients and waste products in each of three MPSs (cardiac, liver, gut; Table 1). Specifically, in a 6-day-long experiment without media change, nutrient dependence and waste accumulation kinetics along with phenotypic metrics for functionality and viability were measured in the three MPSs (Fig. 1). Metabolic activity of the cardiac MPS as measured by PrestoBlue (which measures chemical reduction of non-fluorescent resazurin to fluorescent resorufin in mitochondria^43^) was well maintained throughout the course of the experiment. Functionality of the liver MPS was assessed by albumin secretion rate. The observed rates were consistent with historical liver MPS data from our laboratory^41^, indicating no adverse effects with extended culture duration. As evaluated by TEER measurements, the gut MPS maintained an intact barrier function over the six days, although TEER was continuously decreasing and by day six was near 200 Ohm*cm^2^, which is considered a threshold for impairment of barrier integrity for the Caco-2/HT-29 coculture model in our MPS^5^. These results indicate the possibility of extending cell culture period up to six days with no medium change without considerably altering functionality or viability for the cardiac and liver MPSs and for shorter times for the gut MPS. Fluid loss due to media evaporation was on average less than 11% for all three MPSs after six days (Supplementary Data S1).

**Table 1:** Overview of MPS configuration, MPS-specific hardware and cell parameters.

MPS	Cardiac	Liver	Gut
Cell type	iPSC-derived cardiomyocytes	Human primary hepatocytes	Cancer cell line (differentiated Caco-2/HT-29)
Format	96-well	LiverChip	12-well transwell
Maturation/Differentiation duration post seeding (days)	2	3	21
Seeding density	0.3 × 10^6^/cm^2^	6 × 10^5^/scaffold	0.9 × 10^5^/cm^2^ (9:1 ratio)
Operating media volume (ml)	0.2	2.1	Apical: 0.5 Basal: 1.5
Media volume per seeded cell (nl/cell)	2.1	3.5	17
Media flow	static	recirculation	static
Media change frequency	2 days	2 days	2 days

**Figure 2:**
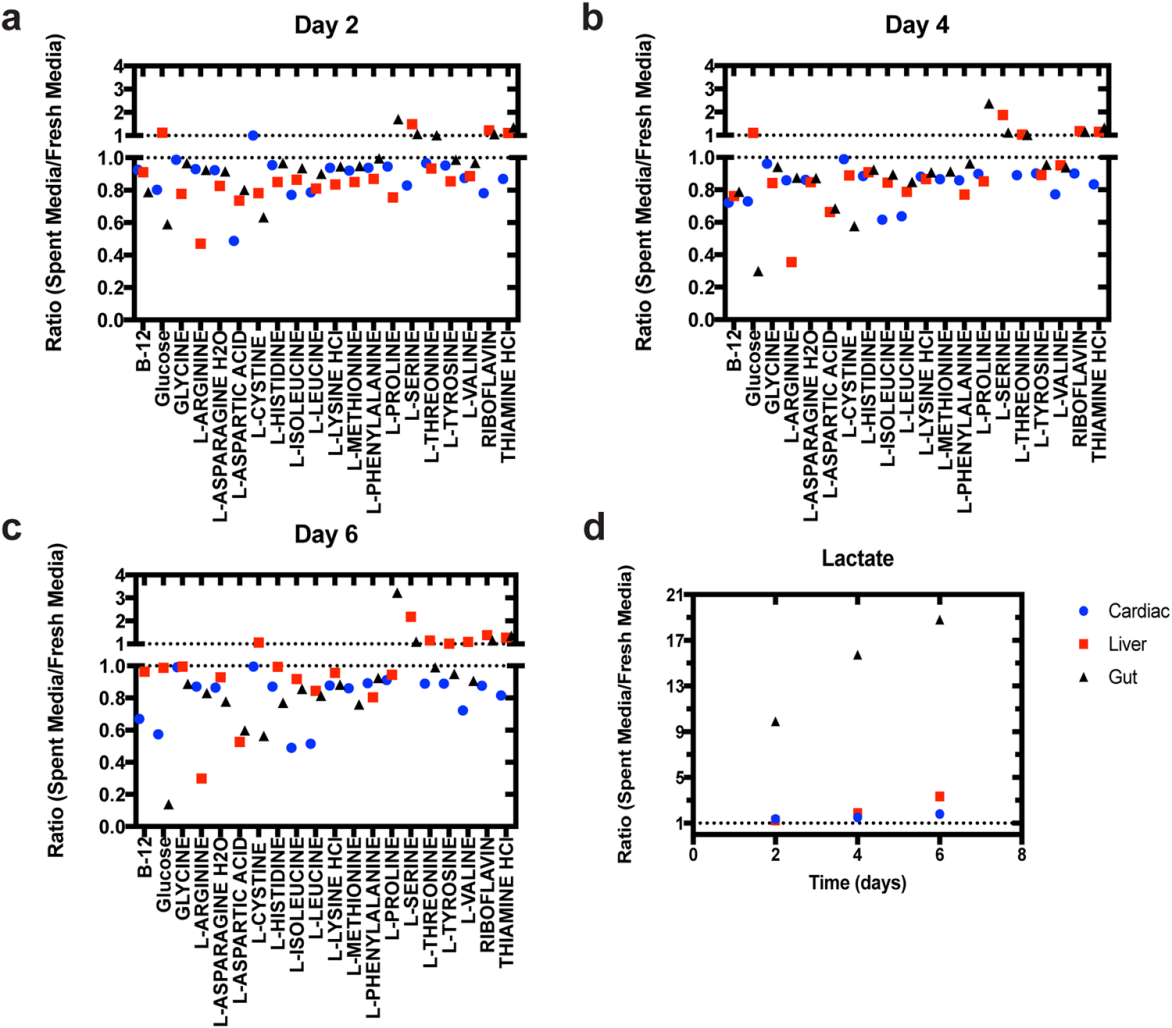
Identifying MPS-specific nutrient needs. Spent media components were profiled for gut MPS, cardiac MPS and liver MPS over six days. Changes in normalized medium component concentrations (spent medium/fresh medium) for all MPSs (black triangles: gut; red squares: liver; blue circles: cardiac; symbols represent average over three biological replicates) were evaluated at day 2 (**a**), day 4 (**b**) and day 6 (**c**). Changing lactate levels were plotted separately for visual purposes (**d**). Concentrations of components in spent media were normalized to fresh media concentrations such that values below 1 indicate net consumption and above 1 indicate net production.

Levels of amino acids and water-soluble vitamins were measured from fresh media and spent media at days 2, 4, and 6 in this experiment (Fig. 2). Normalized media components values, calculated as the ratio of spent over fresh media concentrations, enable an MPS-to-MPS comparison (Fig. 2, absolute values provided in Supplementary Data S2). For the gut MPS, changes in total nutrient amount (basal plus apical) are provided.

For the cardiac MPS, the median normalized concentration values of the media components were: 0.93, 0.87, 0.87, at day 2, 4 and 6, respectively. With the exception of lactate, which accumulated over time, each measured component showed a trend toward additional consumption as culture length increased (observed ranges of the normalized media components: 0.49–1.35, 0.62–1.49, 0.49–1.79 at days 2, 4, and 6, respectively). In comparison, for the liver MPS the median of normalized concentrations were: 0.85, 0.88, 0.99 at days 2, 4, and 6, respectively, with ranges of 0.47–1.49, 0.35–1.89, 0.30–3.33. For the gut MPS, median values of the component concentrations (0.97, 0.93, 0.89) were similar to the other two MPSs. However, ranges of the observed changes in the gut MPS were more substantial (0.59–9.90, 0.30–15.78, 0.14–18.81) due to high glucose consumption and lactate accumulation. Considerable changes were observed in the top three consumed components (normalized concentration <1) at day 6, and differences between the three MPSs were notable (Fig. 2). For the cardiac MPS, the top three consumed nutrients as a fraction of initial concentration were isoleucine (0.49), leucine (0.51) and glucose (0.57); for liver: arginine (0.30), aspartic acid (0.53) and proline (0.94); for gut: glucose (0.14), cysteine (0.56) and aspartic acid (0.60). Interestingly, the data illustrate that for the vast majority of components less than 50% was consumed in any of the MPSs over six days in the same medium. The top three products (normalized concentration > 1) in the three MPSs were, for cardiac: lactate (1.79 at day 6; all other components consumed); for liver: lactate (3.3), serine (2.2) and riboflavin (1.4); for gut: lactate (18.8), proline (3.2) and thiamine (1.4).

Overall, these results demonstrate that limiting nutrient consumption and waste production can be identified, providing critical information needed to rationally optimise operational strategies paired to specific medium formulations.

### Data-driven Quasi-Steady State Operational Strategies

Because the greatest nutrient consumption (glucose) and waste production (lactate) rates were found in the gut MPS, this MPS was selected for a proof of concept study to develop a new media-change strategy that could maintain glucose and lactate concentrations within desirable ranges during extended culture without completely replacing media every two-to-three days.

**Figure 3:**
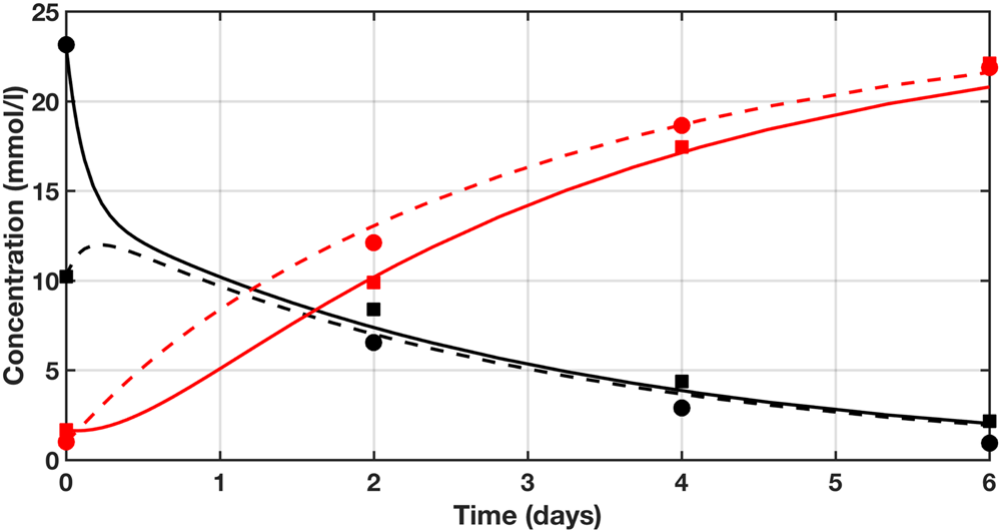
Computational modeling of glucose consumption and lactate production in the gut MPS. Data-driven mechanistic modeling of glucose (black) and lactate (red) in the gut MPS for the 6-day, no medium change experiment. Measurements (symbols) and simulation (lines) of concentrations in the apical side (solid lines; circles) and basal side (dashed lines; squares) of the gut MPS are shown. Triplicates were pooled together due to low apical volumes.

A computational two-compartment model (Supplementary Fig. S1) was developed to describe the consumption of glucose and production of lactate in the gut MPS in a more mechanistic manner, with the compartments representing the apical and basal medium locations. As shown in Fig. 3, the model described the glucose and lactate time-concentration profiles well in the 6-day, no media change, experiment described above when utilizing the glucose consumption and lactate production rate constants estimated from fitting all data points simultaneously: k_gluc_ = 0.4/day for glucose and for lactate k_lac_ = 0.8/day, respectively. For comparison, using analogous models, the rate constants for glucose utilization and lactate production in the liver and cardiac MPSs in the 6 day, no media change, experiment were also calculated as follows: k_gluc_ = 0.02/day (liver) and 0.09/day (cardiac); k_lac_ = 0.06/day (liver) and 0.1/day (cardiac).

We observed greatly elevated lactate concentration (~22 mM) in conjunction with reduced gut TEER in our 6-day no media change experiment (Figs 1 and 3), and hypothesized that lactate could be a major cause of reduced TEER in that experiment. To directly evaluate effects of high lactate concentrations in the gut MPS, exogenous lactate at concentrations of 10, 20, 30 and 40 mM were added to the apical site of the gut MPS and barrier integrity was monitored via TEER measurement at 24 and 48 h after dosing. As indicated in Supplementary Fig. S4, a dose-dependent reduction in TEER was observed as lactate concentration was increased. In particular, exposure to 30 and 40 mM lactate for more than 24 hours led to a considerable decrease in barrier integrity, indicating that a successful media change protocol should limit lactate to lower concentrations.

To identify media change protocols that could maintain quasi-steady state conditions for glucose and lactate, the time-concentration profiles of these components were simulated for a range of possible protocol parameters using the two-compartmental model with the observed glucose consumption and lactate production rates. Conditions for selection of acceptable media change and supplementation protocols were set as follows: (i) physiological glucose concentrations of 4–7 mM^44^ and (ii) lactate concentrations not exceeding 20 mM for the experiment duration. In the simulated protocols, a fraction of the medium in the basal compartment (which is analogous to systemic blood circulation) was removed and replaced with fresh media every 24 hours (0–100% of basal media volume) for a 10-day experiment, and simultaneously, supplemental glucose was added to the apical compartment every 24 hours (0–10 mM; mimicking absorption of glucose from lumen).

**Figure 4:**
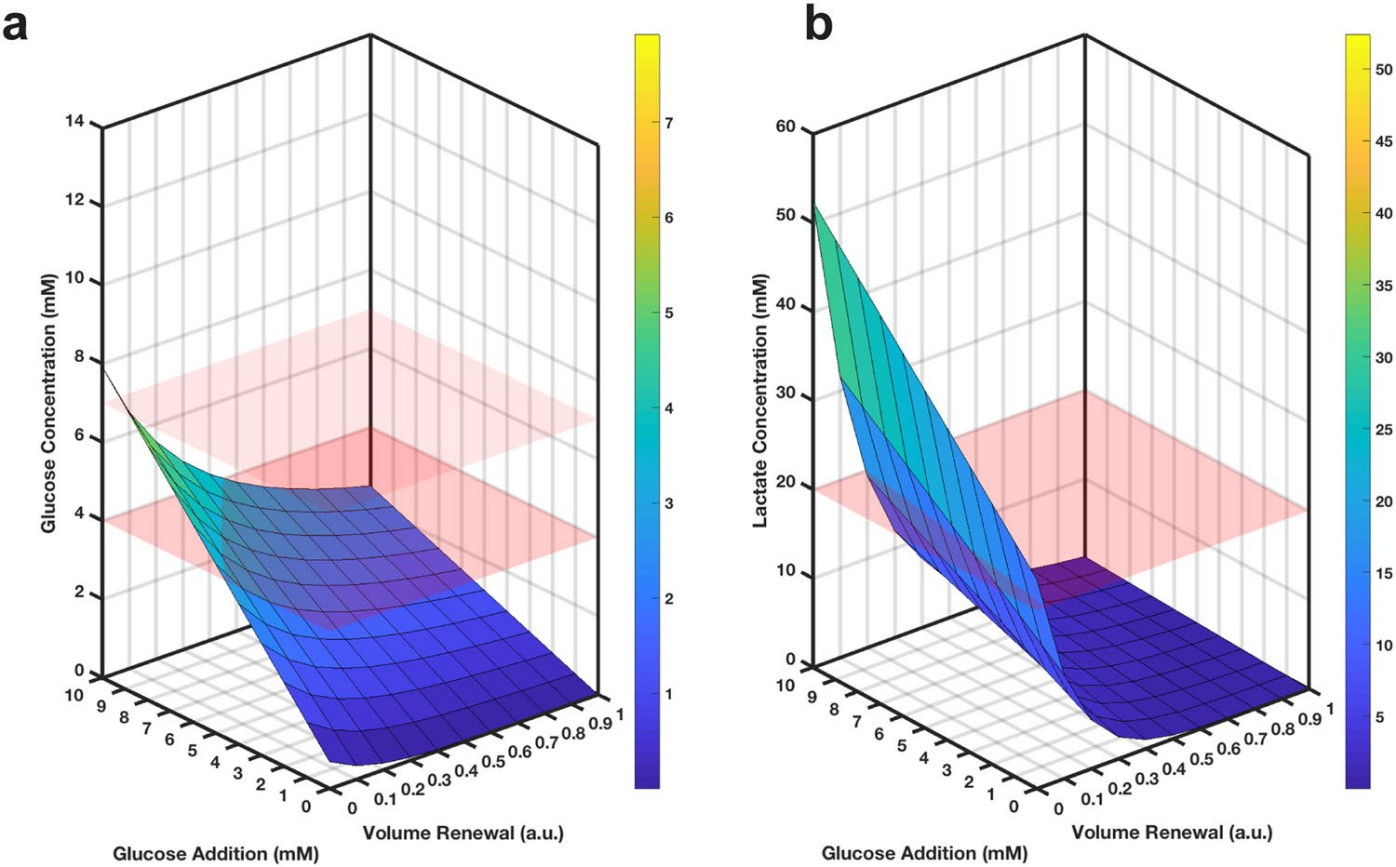
Simulating media change protocols to achieve quasi-steady state operations in the gut MPS. Simulating a range of medium change protocols allows identification of operational strategies that can simultaneously satisfy the desired conditions: (**a**) glucose within physiological levels (between red planes: 4–7 mM) and (**b**) lactate levels below toxic concentrations (below red plane: 20 mM). Media change protocols simulated were replacement of media volume every 24 h from the basal side (0-100%, 10% steps) and supplementing glucose every 24 h to the apical site (0–10 mM, 1 mM steps) for 10 days, with the final glucose and lactate concentrations represented by the simulated surfaces. The color of the simulated surface corresponds to the colorbar on the right-hand side and to values on the z-axis, i.e. represents glucose (**a**) and lactate (**b**) concentrations.

The resulting glucose and lactate concentration profiles at day 10 (apical site) can be seen in Fig. 4. Our specified conditions for glucose and lactate concentrations are satisfied when the simulated surfaces sit between the two red planes in Fig. 4a (glucose) and below the red plane in Fig. 4b (lactate) simultaneously. As seen, there is a small range of acceptable fractional media change and glucose supplementation options when done at 24 h intervals. For example, replenishing 15% of the basal media and simultaneously dosing 6 mM glucose to the apical site of the gut MPS every 24 hours is predicted to maintain the desired glucose and lactate concentrations for up to 10 days.

**Figure 5:**
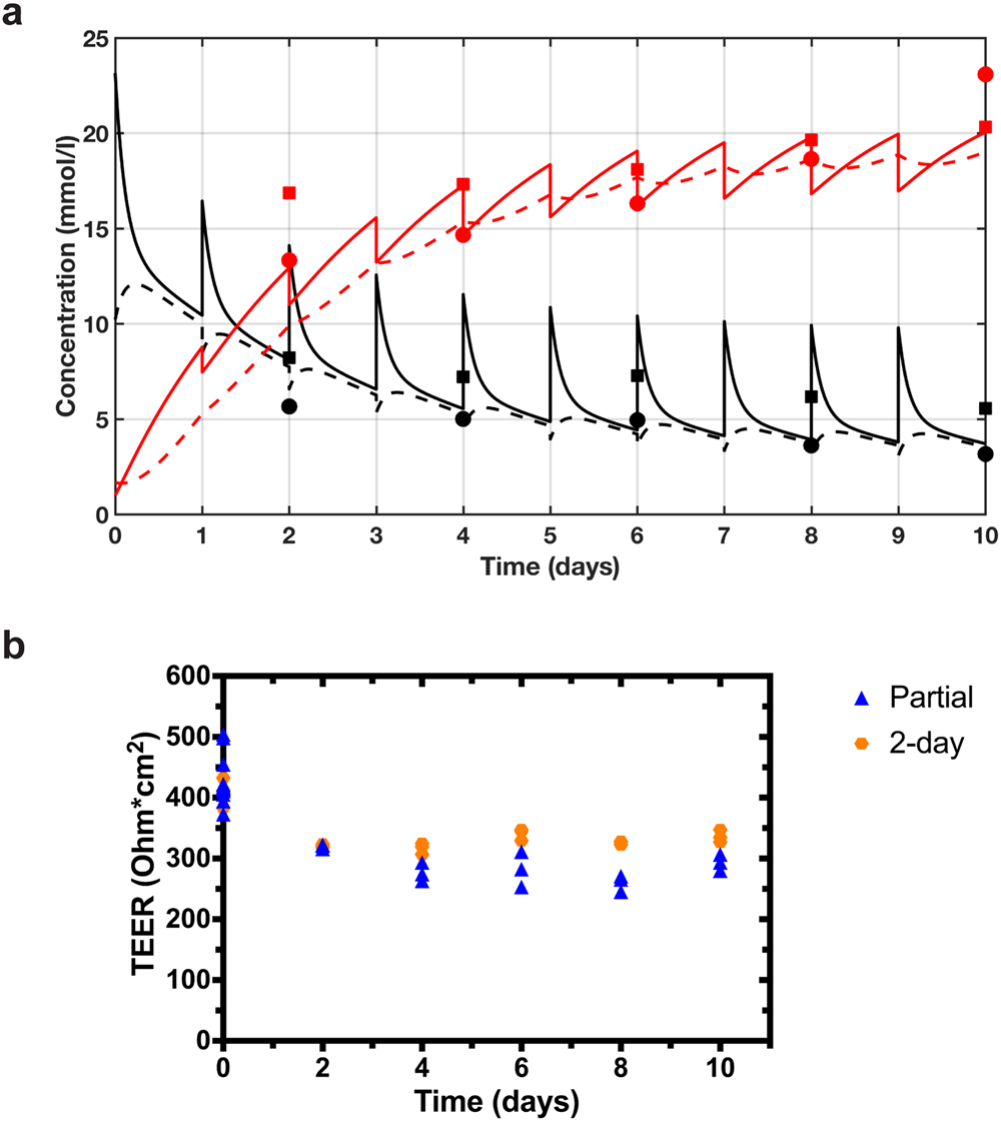
Altered media change protocol for the gut MPS maintains glucose and lactate at steady state levels. Experimental validation of the computationally developed partial media change protocol for the gut MPS is illustrated. (**a**) Predicted quasi-steady state operation maintaining physiological glucose levels in both apical (solid lines) and basal (dashed lines) compartments align well with measured glucose (black symbols) and lactate (red symbols) levels in both compartments (apical: circles; basal: squares). (**b**) Assessment of TEER values showed an intact and comparable barrier function over this extended time-course to the complete 2-day media change. Glucose and lactate concentrations were determined immediately before media replenishment and glucose supplementation.

The ability of this predicted partial media change protocol to maintain glucose and lactate concentrations at quasi-steady state within desired ranges was tested experimentally in the gut MPS. Every 24 h, 15% of the basal media was replaced and apical media was supplemented with 6 mM glucose. Glucose and lactate concentrations were measured in the apical and basal sites at days 2, 4, 6, 8, and 10 (Fig. 5a, symbols), and compared to those simulated from the model (Fig. 5a, curves). Without additional fitting of data, the computational model predicted the effects of the new media change protocol on glucose and lactate concentrations very well (based on the aforementioned estimated rate constants of glucose and lactate from the gut MPS). As predicted, after the first several days, the partial media change protocol maintains a quasi-steady state for glucose and lactate, keeping glucose at physiological concentrations and lactate between 15–20 mM up to day 8 and only modestly exceeding 20 mM at day 10. Importantly, intact barrier function (TEER) was maintained at comparable levels to a separate experiment in which complete media change was done every two days (Fig. 5b). This indicated that the newly defined partial media change strategy has no negative phenotypic impact on the gut MPS functionality over the 10-day course of the experiment.

### Evaluating intracellular effects of different media change protocols

The effects of partial media change protocols on the gut MPS were further assessed with intracellular metabolomics and proteomics. Specifically, the intracellular metabolite and protein expression concentration profiles from the partial media change were compared to profiles from the two-day complete media change.

**Figure 6:**
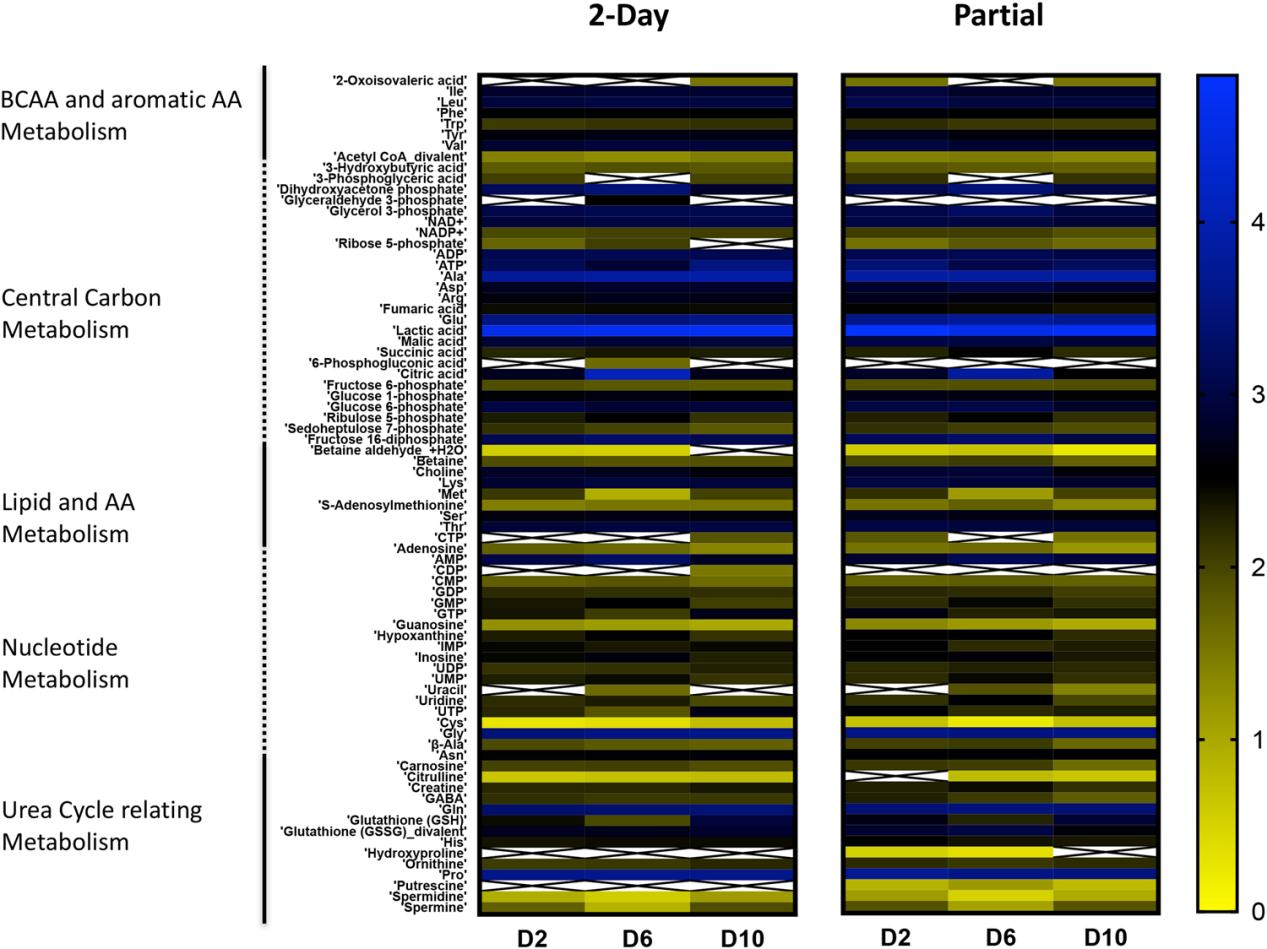
Effect of altered media change protocols on the metabolic fingerprint of the gut MPS. Intracellular metabolite concentrations were quantified for a 2-day media change (left panel) and partial media change (right panel) protocol for the gut MPS at days 2 (D2), 6 (D6) and 10 (D10). Metabolites were grouped according to involvement in particular pathways. Changes in metabolites over time appear to be similar between the media change protocols. Crossed boxes indicate no measurements. The colorbar indicates log2-transformed concentration values.

The comparison of intracellular metabolite concentrations showed similar trends between the conditions and over the course of the experiment (Fig. 6). Metabolites were grouped according to their association to a particular metabolic pathway: branched chain amino acids (BCAA) and aromatic amino acids metabolism, central carbon metabolism, lipid metabolism, amino acid metabolism, nucleotide metabolism, and urea cycle relating metabolism.

In total, 77/110 intracellular metabolites were quantified with this targeted metabolomics approach. To assess changes in metabolite concentrations over time more quantitatively, the ratios of partial media change to two-day complete media change experiments for day 2, 6, and 10 were calculated. Averaged over all metabolites, the ratios were: 0.9 ± 0.2, 1.4 ± 0.4 and 1.0 ± 0.3, for day 2, 6, and 10, respectively (individual metabolite ratios and absolute values are presented in Supplementary Figs S5 and S6, and Data S3). This indicates a modest systemic increase in intracellular metabolites at day 6 for the partial relative to the two-day media change protocol. The ratio at day 6 compared to day 2 and 10 was statistically significant (p < 0.05).

In particular, glucose-1-phosphate and glucose-6-phosphate as well as fructose-1,6-diphosphate and fructose-1-phosphate were about 1.5-fold lower, clearly indicating a downregulation in central carbon metabolism. On the contrary for day 6, all pathways were upregulated on average. For example, beta-alanine, mainly associated with nucleotide metabolism, was about 2-fold higher than found in the control, which aligns well with upregulated uracil, UTP, UDP and uridine concentrations. However, the investigated metabolites seemed to align back to similar levels at day 10 as indicated by an average ratio value of 1.0.

Relative protein abundance for both conditions at day 2, 6, and 10 was assessed between the conditions (Supplementary Figs S5 and S6, and Data S4). In total, 5179 proteins were identified and shared among both conditions and evaluation days. Relative protein abundance levels between the conditions and days were found to be significantly different (p < 0.05, Wilcoxon t-test). To further highlight changes between the conditions, the ratio of relative protein abundance per day found in the partial media change over the two-day media change was calculated and presented in Supplementary Fig. S5 indicating how individual ratios change over time. On average, the ratios are (1.00 ± 0.06, range: 0.55–1.82), (1.02 ± 0.07, range: 0.55–2.00) and (1.01 ± 0.08, range: 0.54–2.09) for day 2, 6 and 10, respectively.

### Operational strategies for integrated multi-MPS platforms

**Figure 7:**
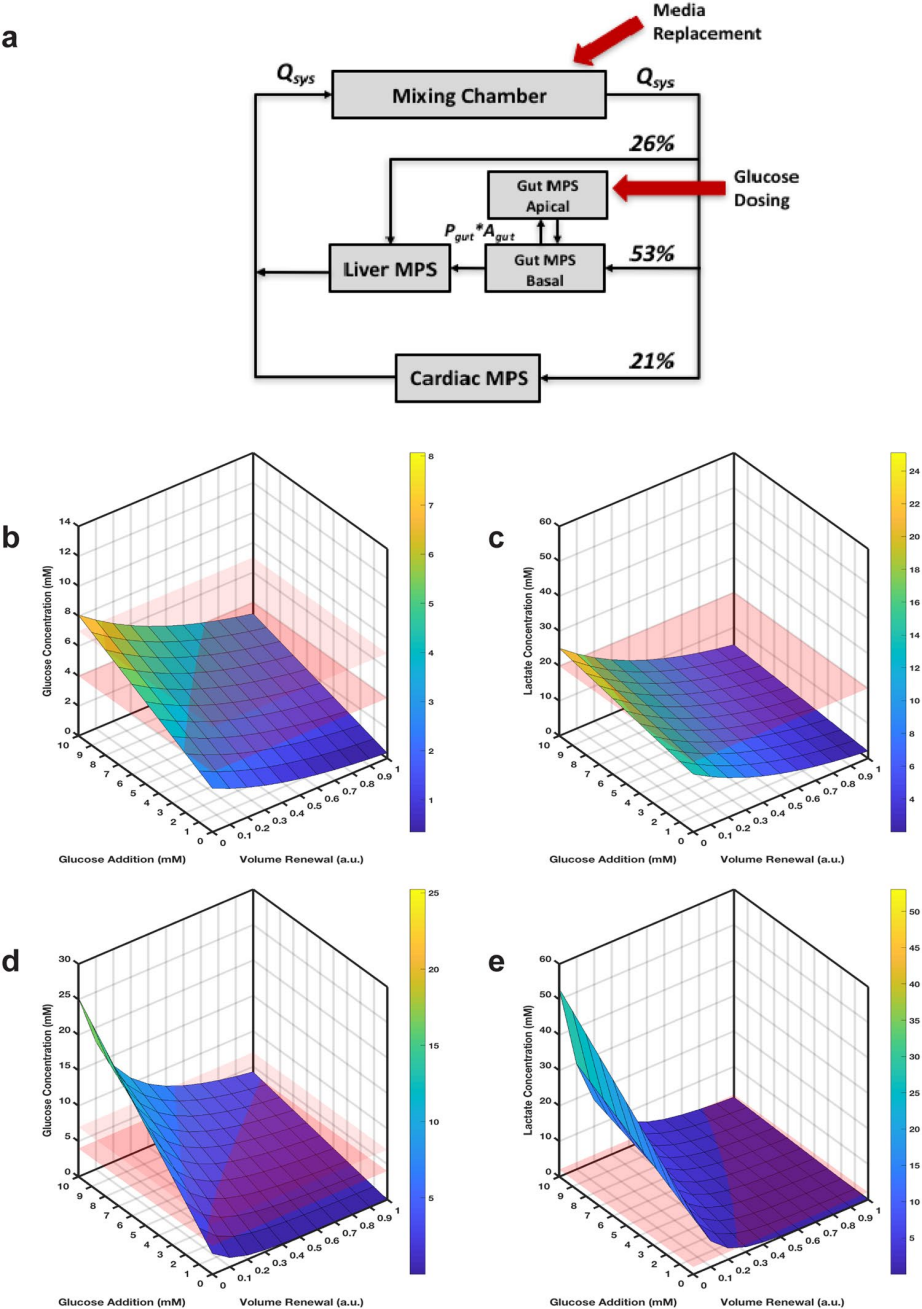
Simulation of quasi-steady state operational media change protocols for an integrated 3-MPS platform. Operational strategies were investigated for an integrated 3-MPS platform (**a**) consisting of gut, liver and cardiac MPSs as well as a mixing chamber to identify protocols that could maintain glucose and lactate concentration within desired ranges. (**b**)  Desired glucose concentration: 4–7 mM (between red planes); (**c**) desired lactate concentration: <20 mM (below red plane). Media change protocols simulated were replacement of media volume every 24 h from the basal site of the gut (0-100%, 10% steps) and supplementation of glucose every 24 h to the apical site of the gut (0–10 mM, 1 mM steps) for 10 days, with the final glucose (**d**) and lactate (**e**) concentrations represented by the simulated surfaces. (**d**) Desired glucose: 4–7 mM (between red planes); (**e**) desired lactate: below 2 mM (physiological levels; below red plane). The range of media change protocols were simulated as in (**b**) and (**c**) except the replacement/replenishment interval was 6 h. The simulated surfaces are the final glucose (left) and lactate (right) concentrations after 10 days. The color of the surfaces in (**b**), (**c**), (**d**), and (**e**) corresponds to the colorbar on the right-hand side and to values on the z-axis, i.e. represents glucose (left, b and d) and lactate (right, c and e) concentrations.

The measured kinetic changes of media components in the three MPSs quantified above were used to identify operational strategies for a theoretically integrated multi-MPS platform consisting of a gut, liver and cardiac MPS. Using a computational model (Supplementary Fig. S3), the time-concentration profiles of glucose and lactate were simulated in a platform in which the three MPSs were connected through a mixing chamber (mimicking a ‘blood’ or ‘systemic circulation’ compartment, Fig. 7a). Similar to the gut MPS investigation above, the goal was to identify media change strategies that could maintain glucose and lactate to between 4–7 mM and below 20 mM, respectively, in the 3-MPS platform for up to 10 days.

To do so, fractional media replacement in the mixing chamber (0–100% of mixing chamber media volume) and glucose addition to the apical site of the gut MPS (0–10 mM) at 6 and 24 h intervals were simulated (Fig. 7b,c,d,e and Supplementary Fig. S3). As for the isolated gut MPS above, a range of operational settings could achieve quasi-steady state operation at the desired conditions (Fig. 7b,c,d,e), including one similar to that used for the isolated gut MPS above: replacing 15% of the medium in the mixing chamber and dosing glucose at 6 mM to the apical site of the gut MPS every 24 h.

For some applications, it may be desirable to maintain lactate at physiologic (1–2 mM), not just nontoxic (< = 20 mM), levels. To achieve this in the 3-MPS platform, a more frequent partial media change was required. Examination of Fig. 7b and c shows there is no media change protocol at 24 h intervals that can meet this condition, while Fig. 7d and e and Supplementary Fig. S7 with partial media change every 6 hours show a range of conditions that can. One protocol that can meet the conditions for up to 10 days is, for example, replacing 60% of mixing chamber volume every 6 hours along with re-dosing 6 mM glucose to the apical site of the gut MPS.

## Discussion

The aim of this work was to characterize the nutrient needs and metabolic landscape of MPSs and use these to design alternative operational strategies for single MPSs and multi-MPS platforms that might better support MPS applications in pharmacology and biology research. The presented approach considered MPS-specific nutrient requirements to inform operational protocols for applications of interest.

Initial profiling of spent media from individual cardiac, liver and gut MPSs over six days revealed distinct patterns of nutrient consumption and waste production. The observed differences among these three constructs likely represent differences in both organ origin and cell source (iPSC-derived cells, human primary cells, and immortalized cancer lines for cardiac, liver, and gut, respectively). The most prominent changes were observed in glucose and lactate concentrations in the gut MPS, defining glucose as a limiting nutrient and lactate as a major accumulated waste product. Interestingly, the vast majority of measured nutrients were less than half consumed in any of the MPSs even after six days with no medium change (Fig. 2), illustrating that most were present in substantial excess for the design of these MPSs, given their specific cell numbers and medium volumes. For the gut MPS, the net glucose consumption and lactate production rates were considerably higher than any components analyzed for the cardiac and liver MPSs, with liver having the lowest rates of all three. While our rate measurements reflect MPS-specific attributes like cell numbers and medium volumes as well as the specific metabolic activity of the different cell types in the three separate MPSs, the latter findings for liver may also reflect the ability of hepatocytes to produce glucose through gluconeogenesis by consuming lactate^45,46^.

The information about MPS-specific nutrient utilization in the gut MPS was used in a computational model of the system to identify a new media change protocol that could provide quasi-steady state nutrient and waste conditions over an extended duration experiment. We specifically aimed to maintain glucose within a physiological range (4–7 mM,^44^) and limit lactate levels to less than 20 mM. From simulations of a range of partial medium replacement and glucose supplementation at 24 h intervals, acceptable ranges of these operating parameters were predicted. A new media change protocol was selected from those ranges and successfully tested in the gut MPS. The results demonstrated that a modest change in medium replacement protocol, from complete change every 48 hours (our standard) to only 15% replacement every 24 h plus glucose supplementation, could support an extended period of quasi-steady state operation, with only modest variations in glucose and lactate occurring between medium replacement/supplementation. Barrier function of the gut MPS was not altered with this partial media change protocol, and metabolomics and proteomics analyses revealed only modest differences between the new partial media change protocol and the standard two-day complete media change protocol. In all, these results build confidence that medium change protocols can be tailored to desired operational conditions and design of new MPS applications.

Another use of the omics-based characterization would be the comparison of this MPS-based culture with human tissue, e.g., a histological sample of human gut, to examine how molecularly similar or different they are. Arguably, such an analysis would allow categorization of MPSs into more or less physiologically relevant systems (as well as define what ‘relevant’ means) as a direct comparison to human physiology.

The gut study was extended on a theoretical basis to an integrated, 3-MPS platform comprising gut, liver and cardiac MPSs plus a mixing chamber. The measured, MPS-specific nutrient needs were used in simulations of a range of partial medium change protocols to identify those that would maintain quasi-steady state levels of glucose at physiological levels and lactate below a toxic threshold (Fig. 7), or alternatively (and much more stringently) keep lactate at physiological levels (1–2 mM,^47^). The results illustrated that the former was readily achievable in the 3-MPS platform with very practical operating protocols (e.g., 15% medium replacement plus glucose supplementation every 24 hr), while the latter was only possible by replacing more medium much more frequently (e.g., 60% every 6 h while re-dosing glucose at 6 mM).

Together with the gut MPS computational and experimental study, these results illustrate how to inform operational parameters based on tissue-specific nutrient and waste removal needs in the context of platform specifications and practical considerations. The specific medium change protocols shown to meet pre-specified conditions depend upon the physical and biological configuration of our MPSs, in particular, the medium volumes in the MPSs and multi-MPS platform, as well as the cell numbers. Changes to any of these would modulate the shape of the simulated surfaces in Figs 4 and 7, thereby providing the acceptable medium change protocols for the reconfigured MPS or multi-MPS platform. The time interval for medium replacement is likewise modulatory of specific results, as seen by comparing simulated concentrations for 24 h or 6 h medium change intervals for the 3-MPS platform.

Clearly, a rational approach to MPS development would be to consider nutrient needs, desirable nutrient conditions (i.e., maintaining specific concentration ranges), options in medium change protocols, and practical limitations of hardware and operation along with all the relevant biological details (cell source, substrate, etc.) during initial MPS design to ensure that the resulting MPS can be operated as needed to support planned applications. The quasi-steady state operations achievable with partial medium change protocols should support longer-term pharmacologic studies in which exposure to drug metabolites over an extended period can be maintained and hence drug pharmacodynamics or toxicity can be evaluated similar to human responses. Similarly, accumulation of tissue-produced biomolecules such as growth factors and cytokines with such MPS operation should better recapitulate the *in vivo* organ microenvironment compared to complete media change protocols. Replacement of 15% of medium per day removes only a fraction of cell-produced mediators and drug metabolites every 2 days rather than 100% as in our previous standard, complete change protocol. Avoiding frequent complete removal of cell-produced paracrine signals is particularly critical for studying crosstalk among MPSs in multi-MPS platforms. Further, better recapitulation of organ microenvironment *in vitro* may also better mimic *in vivo* responses and hence, improve *in vitro-in vivo* translation.

Although there are multiple reports of complete media changes ranging from 1–4 days for single and multi-MPS platforms^1,5,39,41^ as well as a variety of partial media replacement strategies^13,14,42^ used, the rationale and derivation of these protocols are typically unstated. The presented work is the first report to quantitatively investigate MPS-specific nutrient needs and elucidates the application of such knowledge in quasi-steady state operations for single and multi-MPS platforms.

The current approach focused primarily on glucose and lactate kinetics based upon our initial assessment that these were the most consumed and produced of 21 measured soluble medium components across all three MPSs. A possible improvement would be to monitor and include in the analysis additional media components for which specific control is desired. Large molecules in the culture media, such as insulin and transferrin, and additional waste products, such as ammonia and urea, can be assessed in each system to further understand their effects on the tissue function. Similarly, changes in media composition may also affect pH and osmolarity levels. Thus, these metrics may need to be quantified in future studies. Additionally, for pharmacological applications, drug and drug concentrations may be considered in the optimization conditions for medium change protocols. Further, pathway modeling, informed by nutrient utilization data, metabolomics and proteomics, could be applied towards mechanistic understanding of multi-scale MPS-MPS crosstalk and *in vitro* tissue behavior as well as comparison to *in vivo* tissue phenotypes.

One limitation of this study includes the use of a simple first order kinetic model to describe glucose utilization and lactose production rates. More sophisticated models might be needed if clearly non-first-order or time-dependent changes in media components were observed, however, the selected models captured the data sufficiently for the purposes herein.

Experimentally, the next steps would be to demonstrate even longer term (beyond 10 days) maintenance of MPS function using the partial media change protocol, including demonstration of chronic exposure to added drug and drug metabolites, as well as extension to an integrated multi-MPS system. Finally, for alternate media change protocols, we have only considered intermittent partial changes and not continuous fractional change. This is because most MPSs are currently maintained by hand, not through automated medium removal and replacement, necessitating intermittent not continuous partial medium change. Obviously, an analogous “continuous partial medium change” protocol could be identified using the methods described herein, with the expectation of implementing the protocol in an automated medium handling system to, e.g., exchange 15% of the medium over 24 hours.

Understanding metabolic needs of various MPSs, from different cell origins, is essential for developing optimal operational strategies for MPSs that better represent human physiology *in vitro*. The presented approach combining experimental and computational methodologies allows quantification of biological needs in order to devise strategies to better maintain MPSs at physiological nutrient levels while also improving conditions for extended pharmacologic or biological studies. The approach can be readily adapted to any number and combination of MPSs and applied to a wide range of study objectives.

## Materials and Methods

### MPS Preparation

A comprehensive overview of MPS-specific parameters (e.g. hardware, cell types, seeding densities) is presented in Table 1.

#### Gut MPS

The gut MPS comprises a co-culture of Caco-2 and HT29-MTX cells in a confluent monolayer on a 12-well Transwell inserts, with different culture medium on both the apical and basal sides of the monolayer (static, no recirculation). Specifically, gut MPS was prepared as previously described^5^. Briefly, C2BBe1 (clone of Caco-2; ATCC: CRL-2102) and HT29-MTX cells (ATCC: HTB-38) were seeded in a 9:1 ratio onto rat tail collagen I (Corning 354236) coated Transwell Inserts (Corning 3460) at an overall density of 100 K cells/insert. Cells were seeded and maintained in Advanced DMEM (Gibco 12491-015) supplemented with 10% FBS (Atlanta Biologicals S11150), 1 × GlutaMax (Gibco 35050061), 1% Penicillin/Streptomycin (PS) (Gibco 15140148) with 500 µL in the apical compartment and 1.5 mL in the basal compartment, changed every 2 days. After one week, the medium was changed to Advanced DMEM supplemented with ITS cocktail (Roche 11074547001), 1 × GlutaMax, and 1% PS for two additional weeks of maturation.

For routine culture following maturation, cells were maintained in serum-free apical medium consisting of Phenol red-free DMEM (Gibco 31053-028) supplemented with 1 × ITS, 1% Non-Essential Amino Acids (Gibco 15140-148), 1% GlutaMax, and 1% PS and basal medium consisting of Williams’ E medium (Gibco A1217601), Cocktail B (Gibco CM4000), 100 nM hydrocortisone, and 1% PS. For partial media change and glucose modulation experiments, base media was changed to glucose-free DMEM (Gibco A1443001) plus regular supplements. Glucose was weighed and added to media stocks prior to sterile filtering.

Barrier integrity was quantified by TEER using the EVOM2 and the Endohm-12 (World Precision Instruments) at 37 °C.

#### Liver MPS

The liver MPS comprises human primary hepatocytes cultured on the Liverchip^®^ platform (media reciruclation; purchased from CNBio Innovations). The liver MPS was prepared as previously described^5^. Briefly, LiverChips^®^ were assembled and primed with 1% BSA and PS in PBS. Cryopreserved human primary hepatocytes (Thermo Fisher Scientific, HMCPMS) were seeded (6 × 10^5^ cells per scaffold) into rat tail collagen I (BD 354236) coated polystyrene scaffolds (CN Bio Innovations) housed in the LiverChip^®^ in cold seeding medium (Advanced DMEM + Gibco Cocktail A + FBS). After 24 hours, the media was changed to pre-culture media (Advanced DMEM + Gibco Cocktail B). On the third day, the medium was changed to Williams E medium + Gibco Cocktail B + 100 nM hydrocortisone for the duration of the experiment.

Albumin was quantified in the cell culture media using a human-specific ELISA (Bethyl Labs E80-129).

#### Cardiac MPS

The cardiac MPS comprises iCell Cardiomyocytes 2 (Cellular Dynamics International, Inc CMC-100-012-000.5) seeded into human fibronectin coated 96-well plates at a density of 0.3 × 10^6^ cells/cm^2^ in accordance with the manufacturer instructions provided by Cellular Dynamics International, Inc and as previously described^48^. Cardiomyocytes were cultured in plating media (static, no recirculation; CDI CMC-100-010) for 2 days, changed to maintenance media (CDI CMM-100-120) for an additional 2 days, and cultured in Williams E medium + Cocktail B + 100 nM hydrocortisone for the duration of the experiment. Maturation of cardiac tissue was assessed by visual inspection of cardiomyocyte beating frequency. Electrophysiology of the cardiac tissue was not evaluated.

Cardiomyocyte viability was assessed using the PrestoBlue cell viability assay (Thermo Fisher Scientific A13261), an accepted measure of metabolic activity^43^.

### Gut MPS Relative Proteomics

#### Protein Extraction and Digestion

Cell pellets were lysed in lysis buffer [50 mM ammonium bicarbonate, pH 8.5, 0.05% ProteaseMax (Promega, Madison, WI), 1 × protease and phosphatase inhibitor (Cell Signaling Technology, MA)], vortexed and sonicated briefly until solution turned clear. Protein concentrations were determined by BCA protein assay (Thermo-Fisher Scientific, Rockford, IL). Protein samples were reduced with 10 mM TCEP and alkylated with 25 mM iodoacetamide prior to trypsin digestion in 1/50 enzyme/protein ratio at 37 °C overnight. Digests were acidified with formic acid and subjected to Sep-Pak C18 solid phase extraction (Waters, Waltham, MA) and resuspended in 100 µL 50% acetonitrile (ACN)/50% water.

Tandem Mass Tag (TMT) labeling, high pH reverse phase fractionation, and LC-MS analysis were performed by The Danforth Center. The analytical methods are described in detail in the Supplementary Methods S1.

#### Experimental Design and Cell Culture Protocols

Experiments were set up with 3 biological replicates. Experimental design for all three MPSs consisted of parallel running study arms, with takedowns at day 2, 4, and 6, respectively. Additionally, for the gut MPS cell culture media was sampled at day 0, 2, 4, 6, 8, and 10. All experiments were initiated at day 0 after maturation/differentiation period (Table 1). Day 0 of each experiment is defined as 2, 3, and 21 days after cell seeding for the cardiac, liver, and gut MPSs, respectively.

Samples from the apical site of three biological replicates were pooled together due to low media volume.

Cell culture media at these time points, alongside fresh media samples were harvested, centrifuged to remove cellular material, and stored at 4 °C (<7 days) or −20 °C (≥7 days) prior to spent medium analyses. Amino acids, water-soluble vitamins, and other cell culture metabolites were measured via BioProfile FLEX (Nova Biomedical, Waltham, MA) or high/ultrahigh performance liquid chromatography (HPLC/UHPLC). Spent media samples were analysed for amino acid content using Waters AccQ-Tag Ultra Derivatization Kit, AccQ-Tag Ultra column, and AccQ-Tag Ultra eluents using a sample preparation and gradient method modified for cell culture. Samples were analysed for water-soluble vitamin content via HPLC using ion-pairing reverse phase chromatography with a C18 column followed by UV detection.

#### Determining MPS dependency on cell culture media composition

Cell culture media were profiled at day 0, 2, 4 and 6 for all three MPSs (averaging the triplicates) to investigate how nutrients in the media composition are consumed and waste products are produced. To highlight changes in the media composition over time, all components were normalized to the corresponding values at day 0 (fresh media).

A mathematical model was used to determine glucose consumption and lactate production rate constants for all MPSs. For the gut MPS, a two-compartmental model (Supplementary Fig. S1) was used describing the apical (V_apical_ = 0.5 ml) and basal site (V_basal_ = 1.5 ml) of the gut MPS. Simultaneously, the same model was used to monitor lactate concentrations in both compartments. The lactate production rate constant (at the basal site) was assumed to be mostly dependent on the current glucose concentration available in the basal compartment. However, lactate may also be produced from glutaminolysis^49^. Literature values for permeability of glucose and lactate through a Caco-2-monolayer were used^50,51^. The surface area of a 12-well transwell is A = 1.12 cm^2^.

Both rate constants were determined by minimizing the squared difference (least-square) between the model description in both compartments and for both species versus actual measurements of glucose and lactate over time. Similarly, for the liver and cardiac MPS, a one-compartmental model was used and the same workflow was applied to determine glucose and lactate rate constants.

#### Computational investigation of operational strategies

Single-MPS Studies: Operational strategies need to be identified specifically for the application of interest. Working towards more physiologically relevant MPSs and their operations, we aimed at maintaining physiological glucose concentrations (4–7 mM^44^) and simultaneously limiting lactate concentrations to less than 20 mM^52^ in the gut MPS (both apical and basal compartments) over the course of a ten-day experiment. To achieve this, the commonly used 2-day media change was modified. Specifically, fractional media removal from the basal site of the gut MPS (0, 10, 20, …, 100% of basal media volume) as well as glucose dosing to the apical site (0, 1, 2,…, 9, 10 mM) were simulated using the previously estimated glucose and lactate rate constants. Rather than providing fresh media every 48 h, a more frequent protocol was chosen, applying these modifications every 24 h (Supplementary Fig. S2).

This approach allows for the monitoring of glucose and lactate concentrations over time as a function of removing (and replenishing) media as well as providing glucose.

Integrated Multi-MPS Studies: A mechanistic understanding of nutrient consumption and waste production in individual MPSs allows for the integration of this information into a hypothetical multi-MPS platform. The same workflow as described for the single MPS operation was applied to determine operational strategies to maintain glucose and lactate desired concentrations (e.g. glucose between 4–7 mM (physiological range) and lactate <20 mM (below toxic concentrations)).

Specifically, an interacting cardiac-liver-gut system connected through a mixing chamber (‘blood’ compartment, Supplementary Fig. S3) was simulated. A systemic flow rate of Q_sys_ = 50 ml/day was chosen to achieve adequate mixing among the compartments. Individual MPS volumes were used; for the mixing chamber, a volume of V_mix_ = 1 ml was included. Flow partitioning between the MPSs and the mixing chamber was based on human physiology and cardiac output to the respective organs^53,54^, i.e. the liver MPS received Q_liver_ = 0.26 * Q_sys_, the cardiac MPS received Q_cardiac_ = 0.21 * Q_sys_ and the gut MPS received Q_gut_ = 0.53 * Q_sys_.

A range of protocols was simulated, with media removed and replaced from the mixing chamber (0, 10, 20, …, 100% of mixing chamber media volume) and glucose dosed to the apical site of the gut MPS (0, 1, 2,…,9, 10 mM). A 6 and 24 h media change protocol was simulated monitoring glucose and lactate concentrations in the mixing chamber (Supplementary Fig. S3).

#### Data Analysis and Plotting

All simulations and parameter estimations as well as data analyses were performed in Matlab (R2017a, The MathWorks, Inc., Natick, Massachusetts, United States). Plotting was performed in Prism (v. 7.0c, GraphPad Software, La Jolla, California, United States). Wilcoxon t-tests were performed and p-values < 0.05 were considered significant.

The data that support the findings of this study can be found in the Supplementary Data S1, S2, and S3, and are available from the corresponding author upon request.

## Electronic supplementary material

Supplementary Figures S1

Supplementary Methods S1

Supplementary Dataset S1

Supplementary Dataset S2

Supplementary Dataset S3

Supplementary Dataset S4

## References

[1] Domansky K (2010). Perfused multiwell plate for 3D liver tissue engineering. Lab Chip.

[2] Wilmer MJ (2016). Kidney-on-a-Chip Technology for Drug-Induced Nephrotoxicity Screening. Trends Biotechnol.

[3] Marsano A (2016). Beating heart on a chip: a novel microfluidic platform to generate functional 3D cardiac microtissues. Lab Chip.

[4] Wufuer M (2016). Skin-on-a-chip model simulating inflammation, edema and drug-based treatment. Sci Rep.

[5] Chen, W. L. K. *et al*. Integrated gut/liver microphysiological systems elucidates inflammatory inter-tissue crosstalk. *Biotechnol Bioeng*, 10.1002/bit.26370 (2017). 10.1002/bit.26370 PMC5614865 28667746

[6] Brown JA (2015). Recreating blood-brain barrier physiology and structure on chip: A novel neurovascular microfluidic bioreactor. Biomicrofluidics.

[7] Loskill P (2017). WAT-on-a-chip: a physiologically relevant microfluidic system incorporating white adipose tissue. Lab on a Chip.

[8] Bovard D, Iskandar A, Luettich K, Hoeng J, Peitsch MC (2017). Organs-on-a-chip. Toxicology Research and Application.

[9] Haring AP, Sontheimer H, Johnson BN (2017). Microphysiological Human Brain and Neural Systems-on-a-Chip: Potential Alternatives to Small Animal Models and Emerging Platforms for Drug Discovery and Personalized Medicine. Stem Cell Rev.

[10] Mathur A (2015). Human iPSC-based cardiac microphysiological system for drug screening applications. Sci Rep.

[11] Vernetti L (2017). Functional Coupling of Human Microphysiology Systems: Intestine, Liver, Kidney Proximal Tubule, Blood-Brain Barrier and Skeletal Muscle. Sci Rep.

[12] Tsamandouras, N. *et al*. Integrated Gut and Liver Microphysiological Systems for Quantitative *In Vitro* Pharmacokinetic Studies. *The AAPS journal*, 10.1208/s12248-017-0122-4 (2017). 10.1208/s12248-017-0122-4 PMC6540747 28752430

[13] Maschmeyer I (2015). A four-organ-chip for interconnected long-term co-culture of human intestine, liver, skin and kidney equivalents. Lab Chip.

[14] Oleaga C (2016). Multi-Organ toxicity demonstration in a functional human *in vitro* system composed of four organs. Sci Rep.

[15] Xu Z (2016). Design and Construction of a Multi-Organ Microfluidic Chip Mimicking the *in vivo* Microenvironment of Lung Cancer Metastasis. ACS Appl Mater Interfaces.

[16] Lee H (2017). A pumpless multi-organ-on-a-chip (MOC) combined with a pharmacokinetic-pharmacodynamic (PK-PD) model. Biotechnol Bioeng.

[17] Rogal J, Probst C, Loskill P (2017). Integration concepts for multi-organ chips: how to maintain flexibility. Future Science OA.

[18] Bauer S (2017). Functional coupling of human pancreatic islets and liver spheroids on-a-chip: Towards a novel human *ex vivo* type 2 diabetes model. Sci Rep.

[19] Edington, C. D. *et al*. Interconnected Microphysiological Systems for Quantitative Biology and PharmacologyStudies. *Scientific Reports* 8, 10.1038/s41598-018-22749-0 (2018). 10.1038/s41598-018-22749-0 PMC5852083 29540740

[20] Sung JH, Kam C, Shuler ML (2010). A microfluidic device for a pharmacokinetic-pharmacodynamic (PK-PD) model on a chip. Lab Chip.

[21] Skardal, A., Shupe, T. & Atala, A. Organoid-on-a-chip and body-on-a-chip systems for drug screening and disease modeling. *Drug Discov Today*, 10.1016/j.drudis.2016.07.003 (2016). 10.1016/j.drudis.2016.07.003 PMC9039871 27422270

[22] Marx U (2016). Biology-inspired microphysiological system approaches to solve the prediction dilemma of substance testing. ALTEX.

[23] Yu J (2015). Quantitative Systems Pharmacology Approaches Applied to Microphysiological Systems (MPS): Data Interpretation and Multi-MPS Integration. CPT Pharmacometrics Syst Pharmacol.

[24] Maass C, Stokes CL, Griffith LG, Cirit M (2017). Multi-Functional Scaling Methodology for Translational Pharmacokinetic and Pharmacodynamic Applications using Integrated Microphysiological Systems (MPS). Integrative Biology.

[25] Luni C, Serena E, Elvassore N (2014). Human-on-chip for therapy development and fundamental science. Curr Opin Biotechnol.

[26] Caballero D (2017). Organ-on-chip models of cancer metastasis for future personalized medicine: From chip to the patient. Biomaterials.

[27] Weltin A (2017). Accessing 3D microtissue metabolism: Lactate and oxygen monitoring in hepatocyte spheroids. Biosens Bioelectron.

[28] Stokes CL, Cirit M, Lauffenburger DA (2015). Physiome-on-a-Chip: The Challenge of “Scaling” in Design, Operation, and Translation of Microphysiological Systems. CPT Pharmacometrics Syst Pharmacol.

[29] Eagle H (1955). Nutrition Needs of Mammailan Cells in Tissue Culture. Science.

[30] Birsoy K (2014). Metabolic determinants of cancer cell sensitivity to glucose limitation and biguanides. Nature.

[31] Favaro E (2012). Glucose utilization via glycogen phosphorylase sustains proliferation and prevents premature senescence in cancer cells. Cell Metab.

[32] Schug ZT (2015). Acetyl-CoA synthetase 2 promotes acetate utilization and maintains cancer cell growth under metabolic stress. Cancer Cell.

[33] Reinhart D, Damjanovic L, Kaisermayer C, Kunert R (2015). Benchmarking of commercially available CHO cell culture media for antibody production. Appl Microbiol Biotechnol.

[34] Miller WM, Wilke CR, Blanch HW (1989). The transient responses of hybridoma cells to nutrient additions in continuous culture: II. Glutamine pulse and step changes. Biotechnology and Bioengineering.

[35] Miller WM, Wilke CR, Blanch HW (1989). Transient responses of hybridoma cells to nutrient additions in continuous culture: I. Glucose pulse and step changes. Biotechnology and Bioengineering.

[36] Schmid G, Blanch HW, Wilke CR (1990). Hybridoma growth, metabolism, and product formation in HEPES-buffered medium: II. Effect of pH. Biotechnology Letters.

[37] Meadows AL (2008). Metabolic and Morphological Differences between Rapidly Proliferating Cancerous and Normal Breast Epithelial Cells. Biotechnology Progress.

[38] Materne, E. M. *et al*. The multi-organ chip–a microfluidic platform for long-term multi-tissue coculture. *J Vis Exp*, e52526, 10.3791/52526 (2015). 10.3791/52526 PMC4541596 25992921

[39] Miller, P. G. & Shuler, M. L. Design and demonstration of a pumpless 14 compartment microphysiological system. *Biotechnol Bioeng*, 10.1002/bit.25989 (2016). 10.1002/bit.25989 27070809

[40] Kim JY (2015). 3D spherical microtissues and microfluidic technology for multi-tissue experiments and analysis. J Biotechnol.

[41] Tsamandouras N (2017). Quantitative Assessment of Population Variability in Hepatic Drug Metabolism Using a Perfused Three-Dimensional Human Liver Microphysiological System. J Pharmacol Exp Ther.

[42] Wagner I (2013). A dynamic multi-organ-chip for long-term cultivation and substance testing proven by 3D human liver and skin tissue co-culture. Lab Chip.

[43] Chen JL, Steele TWJ, Stuckey DC (2018). Metabolic reduction of resazurin; location within the cell for cytotoxicity assays. Biotechnol Bioeng.

[44] Santiago JV, Clarke WL, Shah SD, Cryer PE (1980). Epinephrine, Norepinephrine, Glucagon, and Growth Hormone Release in Association with Physiological Decrements in the Plasma Glucose Concentration in Normal and Diabetic Man*. The Journal of Clinical Endocrinology & Metabolism.

[45] Rui L (2014). Energy metabolism in the liver. Compr Physiol.

[46] Bissell DM, Levine GA, Bissell MJ (1978). Glucose metabolism by adult hepatocytes in primary culture and by cell lines from rat liver. Am J Physiol.

[47] Andersen LW (2013). Etiology and therapeutic approach to elevated lactate levels. Mayo Clin Proc.

[48] Doherty KR (2013). Multi-parameter *in vitro* toxicity testing of crizotinib, sunitinib, erlotinib, and nilotinib in human cardiomyocytes. Toxicol Appl Pharmacol.

[49] Young, J. D. Metabolic flux rewiring in mammalian cell cultures. *Current opinion in biotechnology* **24**, 10.1016/j.copbio.2013.04.016 (2013). 10.1016/j.copbio.2013.04.016 PMC3775942 23726154

[50] Sun D (2002). Comparison of human duodenum and Caco-2 gene expression profiles for 12,000 gene sequences tags and correlation with permeability of 26 drugs. Pharm Res.

[51] Lam WK, Felmlee MA, Morris ME (2010). Monocarboxylate transporter-mediated transport of gamma-hydroxybutyric acid in human intestinal Caco-2 cells. Drug Metab Dispos.

[52] Ozturk SS, Riley MR, Palsson BO (1991). Effects of Ammonia and Lactate on Hybridoma Growth, Metabolism, and Antibody Production. Biotechnol Bioeng.

[53] Valentin J (2002). Basic Anatomical and Physiological Data for Use in Radiological Protection: Reference Values. Ann ICRP..

[54] Brown RP, Delp MD, Lindstedt SL, Rhomberg LR, Beliles RP (1997). Physiological Parameter Values for Physiologically Based Pharmacokinetic Models. Toxicol Ind Health.

