# Supplementary material for: Establishing quasi-steady state operations of microphysiological systems (MPS) using tissue-specific metabolic dependencies
Source: Sci Rep. 2018 May 22;8:8015. doi: 10.1038/s41598-018-25971-y (PMC5964119; doi:10.1038/s41598-018-25971-y)
Supplement: Supplementary file 1 — Supplementary Figures S1 [file 41598_2018_25971_MOESM1_ESM.pdf]

Establishing quasi-steady state operations of microphysiological systems (MPS) using tissue-specific metabolic dependencies

Authors: Christian Maass[1], Matthew Dallas[2], Matthew E. LaBarge[1], Michael Shockley[1], Jorge Valdez[1], Emily Geishecker[1], Cynthia L. Stokes[3], Linda G. Griffith[1], Murat Cirit[1]*

1 Department of Biological Engineering, Massachusetts Institute of Technology, Cambridge, MA, USA

2 Thermo Fisher Scientific, Frederick, MD, USA

3 Stokes Consulting, Redwood City, CA, USA

*Corresponding Author: Murat Cirit (mcirit@mit.edu)

Supplemental Figures

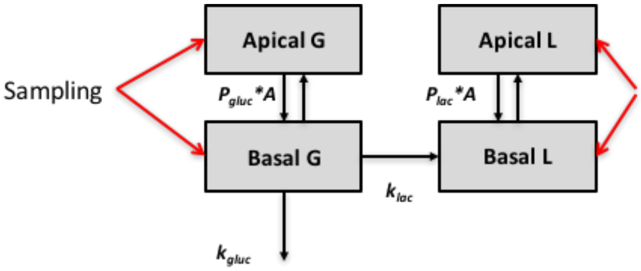

k_{gluc} – Consumption rate	[1/day]	G – Glucose
k_{lac} – Production rate	[1/day]	L – Lactate
P – Permeability (from literature)	[cm/min]	
A – Surface area of gut MPS	[cm^2]	

Fig. S1. Schematic overview of glucose and lactate distribution, consumption and production in the gut MPS. Red arrows indicate media sampling at day 0, 2, 4 and 6.

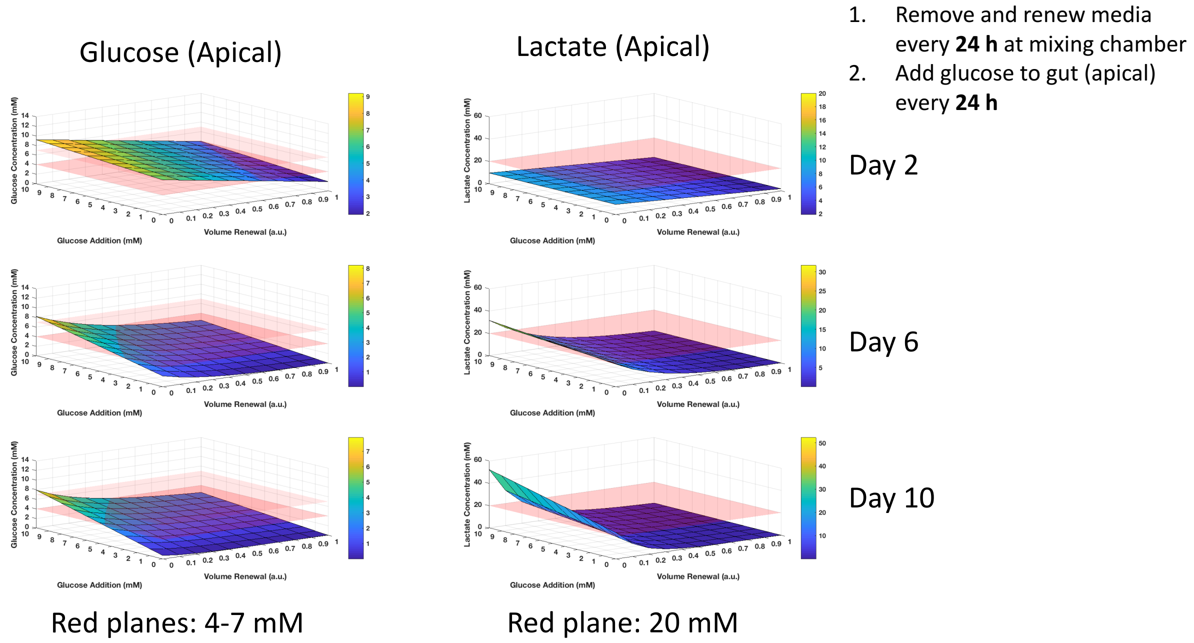

Fig. S3. Identification of optimal operational strategies in the gut MPS for a specific application, i.e. maintaining physiological glucose levels (left panel, red planes: 4-7 mM) and simultaneously limiting lactate to less than 20 mM (right panel, red plane). To achieve this, fractional media changes from the basal site were simulated (0-100%, 10% steps) and glucose additions to the apical site (0-10 mM, 1 mM steps). Removing 15% media and dosing 6 mM glucose every 24 h seemed to satisfy these conditions. The color of the plane corresponds to the colorbar on the right-hand side and to values on the z-axis, i.e. represents glucose and lactate concentrations, respectively.

a)

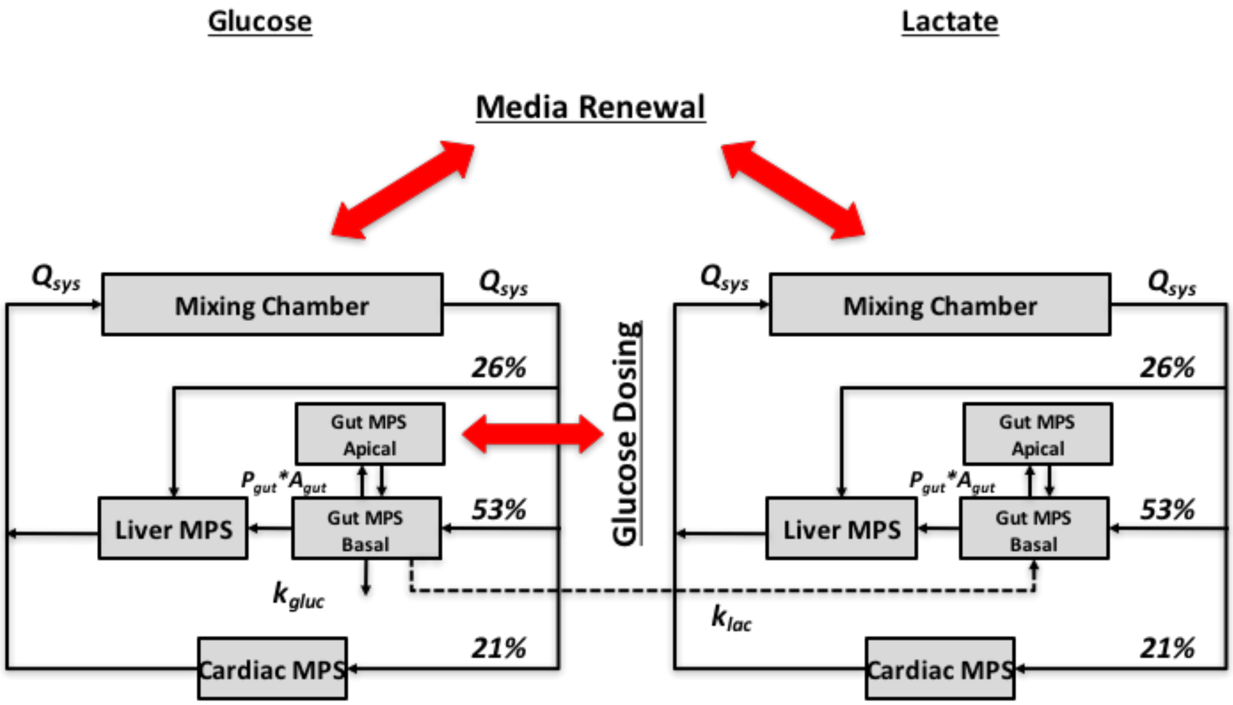
Glucose
Lactate
Media Renewal
Glucose Dosing
Qsys
Mixing Chamber
Qsys
26%
Gut MPS Apical
Pgut*Agut
Liver MPS
Gut MPS Basal
53%
kgluc
klac
Cardiac MPS
21%

b)

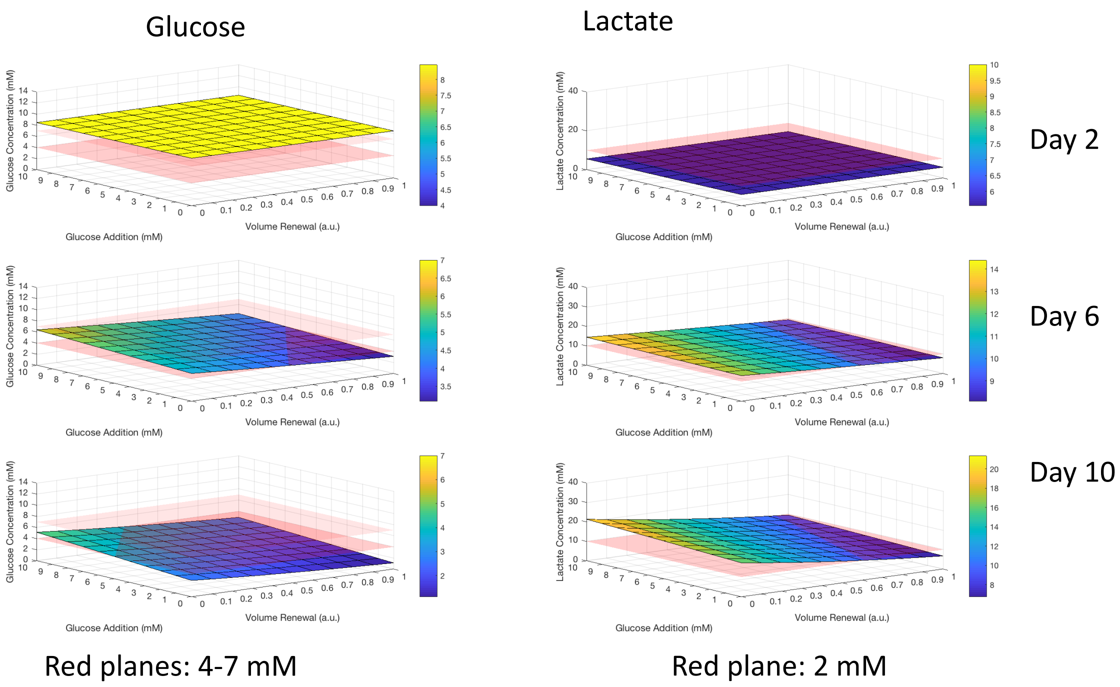
Glucose
Lactate
Day 2
Day 6
Day 10
Glucose Concentration (mM)
Lactate Concentration (mM)
Glucose Addition (mM)
Volume Renewal (a.u.)
Red planes: 4-7 mM
Red plane: 2 mM

Fig. S4. Identification of operational strategies for a simulated integrated multi-MPS platform (a) consisting of a gut, liver and cardiac MPS as well as a mixing chamber ('blood' compartment). More physiological relevant levels of glucose and lactate over the course of 10 days in this interacting system can be achieved by removing e.g. 60% media from the mixing chamber and adding 6 mM glucose to the apical site of the gut MPS every 6 h (b). The color of the plane corresponds to the colorbar on the right-hand side and to values on the z-axis, i.e. represents glucose and lactate concentrations, respectively.

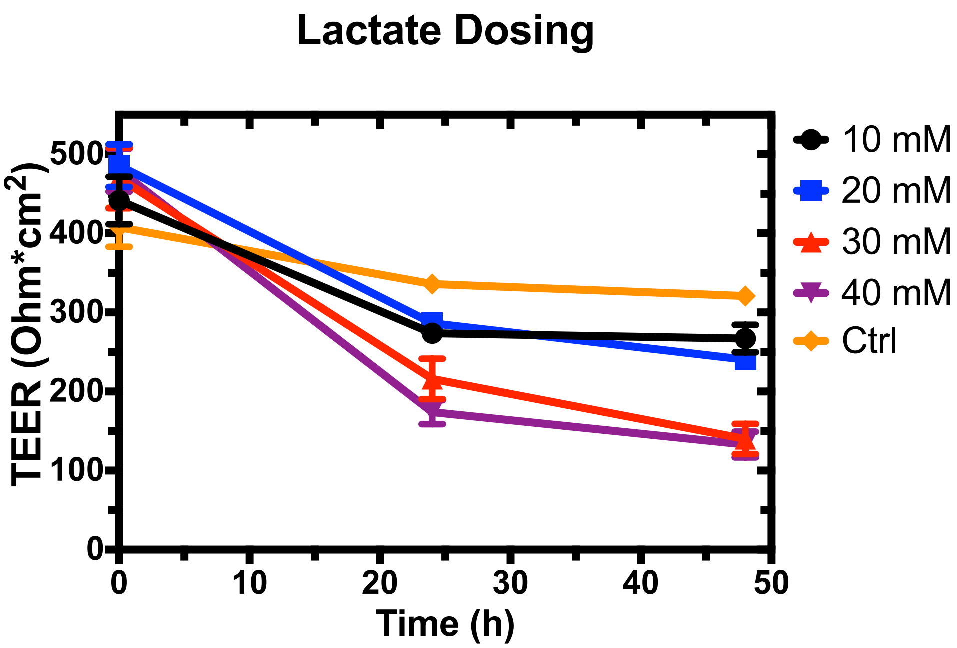

Fig. S5. Dosing exogenous lactate to the gut MPS reveals limiting concentrations of more than 20 mM and an exposure over 48 h as indicated by a considerable decline in barrier function (TEER).

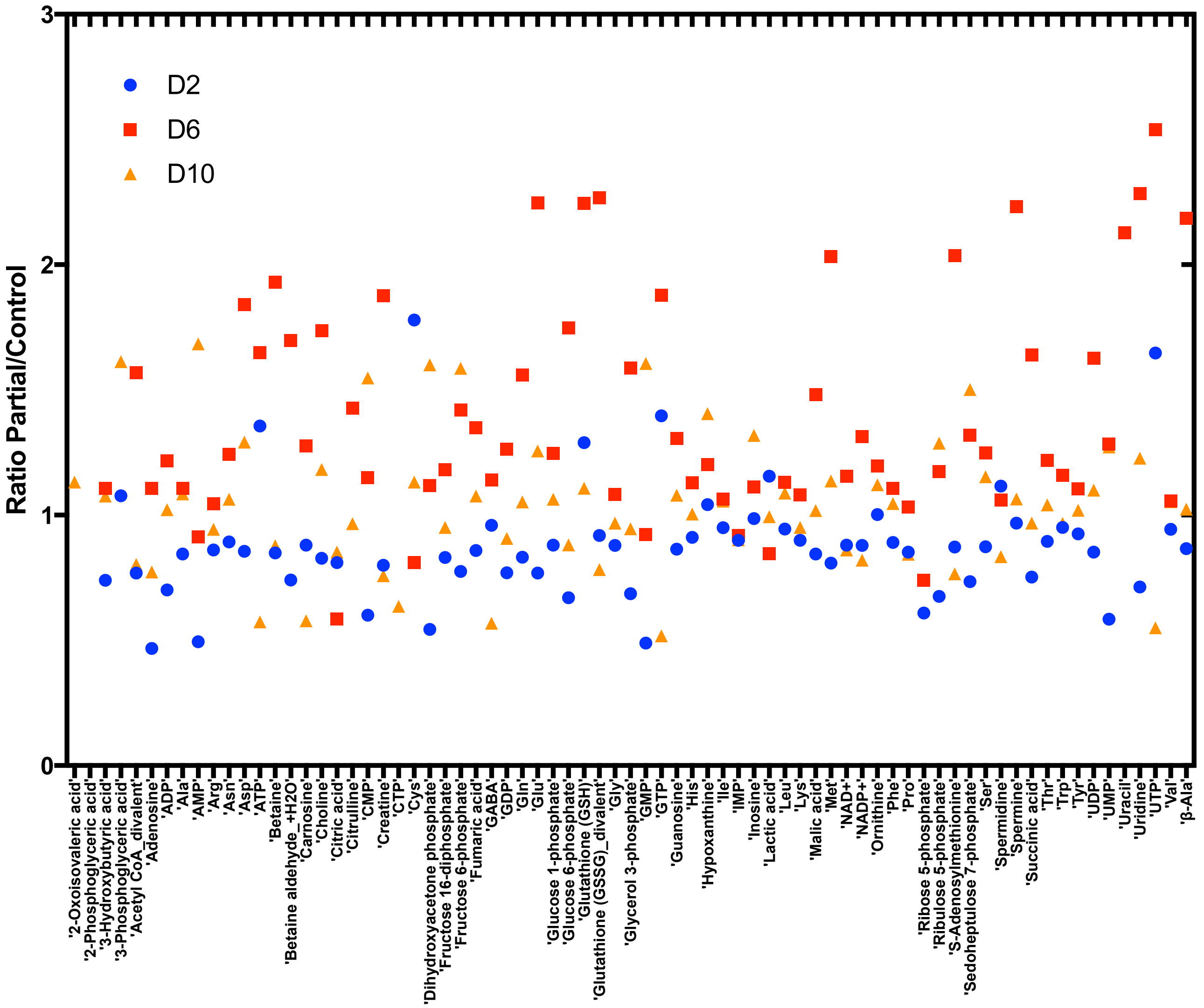

Fig. S6. Metabolic profiling reveals minor changes in metabolites of the gut MPS for the newly developed partial media change protocol and a two-day media change protocol.

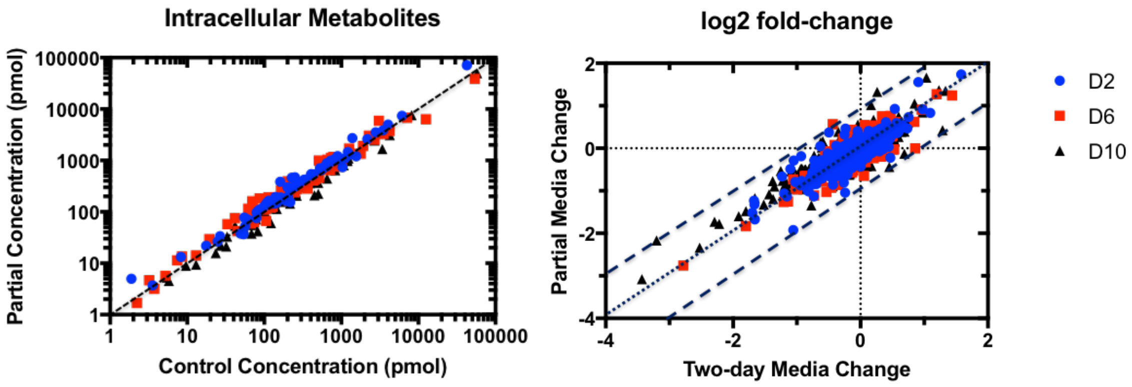

Fig. S7. Actual concentrations of identified metabolites for the 2-day media change (left panel, x-axis) and partial media change (left panel, y-axis). Evidently, there is a strong linear relationship between the conditions and the evaluated times as most of the metabolites are scattered around the line of identity (dashed line); Relationship of relative protein abundance between the 2-day and partial media change evaluated at day 2, 6, and 10 (right panel). Protein expression changes in a similar way for both conditions indicated by the close clustering around the line of identity (dotted line). All changes are within two-fold up- or down regulation (dashed lines).

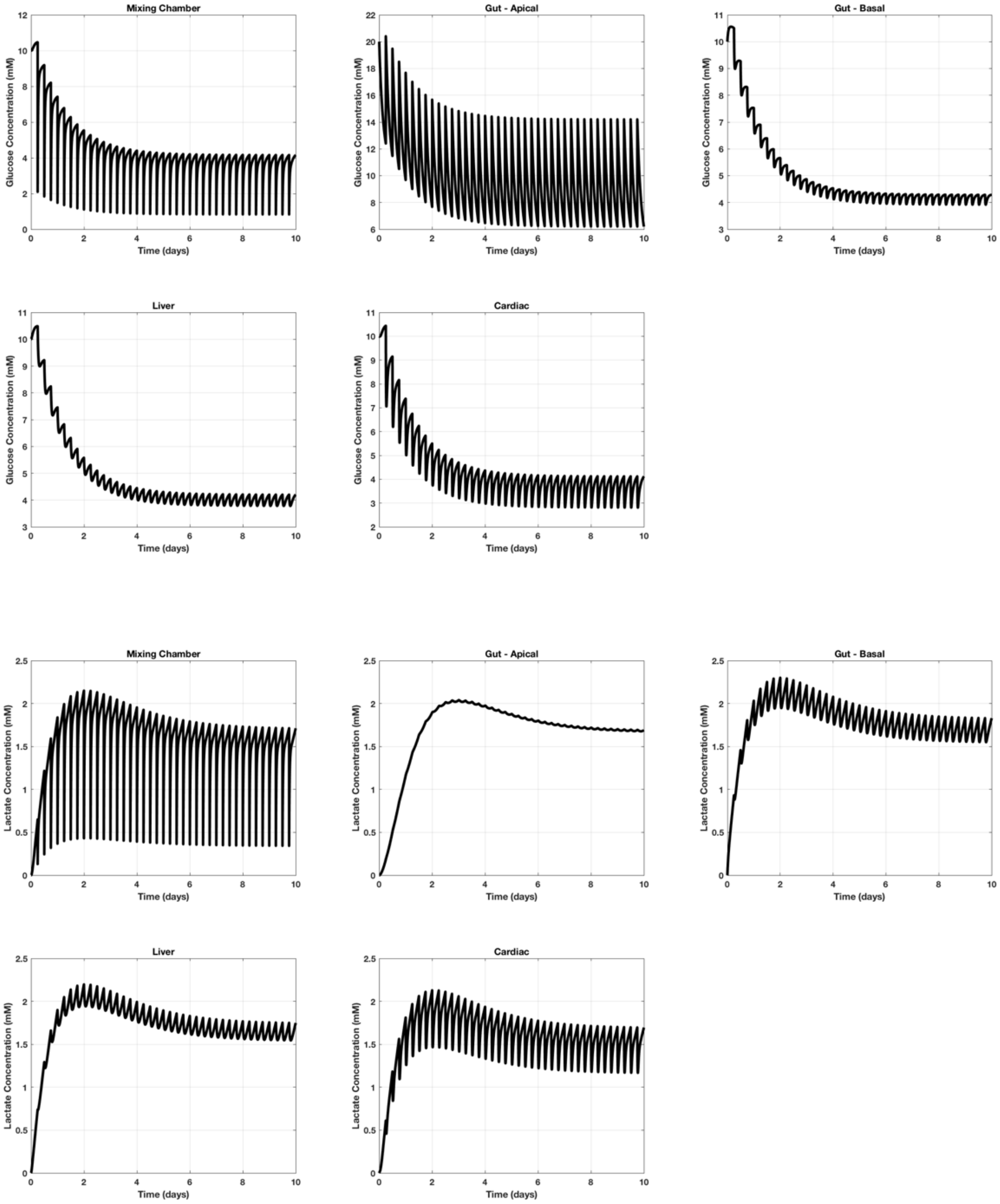

Fig. S8. Simulated glucose (top) and lactate (bottom) time-concentration profiles for the integrated multi-MPS platform. The partial media change protocol consists of removing and replenishing 60% of the media volume in the mixing chamber as well as glucose redosing to the apical site of

the gut MPS at 6 mM every 6 h. This allowed operation of the system at quasi-steady state concentrations of physiologically relevant glucose and lactate levels.
