# Supplementary material for: Establishing quasi-steady state operations of microphysiological systems (MPS) using tissue-specific metabolic dependencies
Source: Sci Rep. 2018 May 22;8:8015. doi: 10.1038/s41598-018-25971-y (PMC5964119; doi:10.1038/s41598-018-25971-y)
Supplement: Supplementary file 2 — Supplementary Methods S1 [file 41598_2018_25971_MOESM2_ESM.pdf]

Establishing quasi-steady state operations of microphysiological systems (MPS) using tissue-specific metabolic dependencies

Authors: Christian Maass[1], Matthew Dallas[2], Matthew E. LaBarge[1], Michael Shockley[1], Jorge Valdez[1], Emily Geishecker[1], Cynthia L. Stokes[3], Linda G. Griffith[1], Murat Cirit[1]*

1 Department of Biological Engineering, Massachusetts Institute of Technology, Cambridge, MA, USA

2 Thermo Fisher Scientific, Frederick, MD, USA

3 Stokes Consulting, Redwood City, CA, USA

*Corresponding Author: Murat Cirit (mcirit@mit.edu)

<u>Computational Model Details</u>

<u>Single MPS</u>

Estimation of rate constants for glucose consumption and lactate production was performed for each MPS individually. The following equations were implemented and solved in Matlab (R2017a, The MathWorks, Inc., Natick, Massachusetts, United States).

1. **Liver and cardiac MPS**

   Glucose

$$\frac{dc_{gluc}(1)}{dt} = \frac{-k_{gluc} * c_{gluc}(1)}{V} \tag{1}$$

   Lactate

$$\frac{dc_{lac}(2)}{dt} = \frac{k_{lac} * c_{gluc}(1)}{V} \tag{2}$$

2. **Gut MPS**

   Glucose

$$\frac{dc_{gluc}(1)}{dt} = \frac{P_{gluc} * A_{gut} * \left(c_{gluc}(2) - c_{gluc}(1)\right)}{V_{gut,ap}} \tag{1}$$

$$\frac{dc_{gluc}(2)}{dt} = \frac{P_{gluc} * A_{gut} * \left(c_{gluc}(1) - c_{gluc}(2)\right) - k_{gluc} * c_{gluc}(2)}{V_{gut,bas}} \tag{2}$$

   Lactate

$$\frac{dc_{lac}(3)}{dt} = \frac{P_{lac} * A_{gut} * \left(c_{lac}(4) - c_{lac}(3)\right)}{V_{gut,ap}} \tag{3}$$

$$\frac{dc_{lac}(4)}{dt} = \frac{P_{lac} * A_{gut} * \left(c_{lac}(3) - c_{lac}(4)\right) + k_{lac} * c_{gluc}(2)}{V_{gut,bas}} \quad (4)$$

where $c_{gluc}(i)$ and $c_{lac}(i)$ are concentrations at time t for each compartment and glucose and lactate, respectively; k describes the rate constant for either glucose consumption or lactate production, P_{gluc} and P_{lac} are the permeability coefficient between the apical and basolateral site of the gut for glucose and lactate, respectively; A_{gut} is the surface area between the gut compartments and V_i describes the volumes of each compartment.

Table 1. Overview of computational model parameters. Glucose and lactate time-concentration profiles were used to determine rate constants. Detailed information about the MPSs can be found in the following references (1-3).

Name	Parameter	Unit	MPS		
			Cardiac	Liver	Gut
Permeability G	P_{gluc}	cm/min	-	-	2E-3 (4)
Permeability L	P_{lac}	cm/min	-	-	5E-4 (5)
Surface Area	A	cm^2	-	-	1.12
Volume	V	ml	0.2	1.6	Apical: 0.5<br>Basal: 1.5

Integrated multi-MPS platform

Using the afore mentioned rate constants, the distribution of glucose and lactate was simulated in an integrated multi MPS platform.

The following equations were implemented and solved in Matlab (R2017a, The MathWorks, Inc., Natick, Massachusetts, United States).

Glucose

Gut MPS

$$\frac{dc_{gluc}(1)}{dt} = \frac{P_{gluc} * A_{gut} * \left(c_{gluc}(2) - c_{gluc}(1)\right)}{V_{gut,ap}} \quad (1)$$

$$\frac{dc_{gluc}(2)}{dt} = \frac{P_{gluc} * A_{gut} * \left(c_{gluc}(1) - c_{gluc}(2)\right) + Q_{gut} * \left(c_{gluc}(5) - c_{gluc}(2)\right) - k_{gluc,gut} * c_{gluc}(2)}{V_{gut,bas}} \quad (2)$$

Liver MPS

$$\frac{dc_{gluc}(3)}{dt} = \frac{Q_{gut} * c_{gluc}(2) + Q_{liv} * c_{gluc}(5) - (Q_{liv} + Q_{gut}) * c_{gluc}(3) - k_{gluc,liv} * c_{gluc}(3)}{V_{liv}} \quad (3)$$

Cardiac MPS

$$\frac{dc_{gluc}(4)}{dt} = \frac{Q_{car} * \left(c_{gluc}(5) - c_{gluc}(4)\right) - k_{gluc,car} * c_{gluc}(4)}{V_{car}} \quad (4)$$

Mixer

$$\frac{dc_{gluc}(5)}{dt} = \frac{Q_{gut} * c_{gluc}(2) + Q_{liv} * c_{gluc}(3) + Q_{car} * c_{gluc}(4) - Q_{mix} * c_{gluc}(5)}{V_{mix}} \quad (5)$$

Lactate
Gut MPS

$$\frac{dc_{lac}(6)}{dt} = \frac{P_{lac} * A_{gut} * \left(c_{lac}(7) - c_{lac}(6)\right)}{V_{gut,ap}} \quad (6)$$

$$\frac{dc_{lac}(7)}{dt} = \frac{P_{lac} * A_{gut} * \left(c_{lac}(6) - c_{lac}(7)\right) + Q_{gut} * \left(c_{lac}(10) - c_{lac}(7)\right) + k_{lac,gut} * c_{gluc}(2)}{V_{gut,bas}} \quad (7)$$

Liver MPS

$$\frac{dc_{lac}(8)}{dt} = \frac{Q_{gut} * c_{lac}(7) + Q_{liv} * c_{lac}(10) - (Q_{liv} + Q_{gut}) * c_{lac}(8) + k_{lac,liv} * c_{gluc}(3)}{V_{liv}} \quad (8)$$

Cardiac MPS

$$\frac{dc_{lac}(9)}{dt} = \frac{Q_{car} * \left(c_{lac}(10) - c_{lac}(9)\right) + k_{lac,car} * c_{gluc}(4)}{V_{car}} \quad (9)$$

Mixer

$$\frac{dc_{lac}(10)}{dt} = \frac{Q_{gut} * c_{lac}(7) + Q_{liv} * c_{lac}(8) + Q_{car} * c_{lac}(9) - Q_{mix} * c_{lac}(10)}{V_{mix}} \quad (10)$$

where $c_{gluc}(i)$ and $c\mathit{lac}(i)$ are the concentrations at time t for every compartment for glucose and lactate, respectively; P_{gluc} and P_{lac} are the permeability coefficients between the apical and basolateral site of the gut for glucose and lactate, respectively; A_{gut} is the surface area between the gut compartments, Q_i refers to the flow and V_i to the volumes of each compartment.

Table 2. Overview of computational model parameters for the simulation of glucose and lactate time-concentration profiles for an integrated multi-MPS platform consisting of cardiac, liver, gut MPSs and a mixing chamber. Detailed information about the MPSs can be found in these references (1-3).

Name	Parameter	Unit	MPS			
			Cardiac	Liver	Gut	Mixer
Permeability Glucose	P_{gluc}	cm/min	-	-	2E-3 (4)	-
Permeability Lactate	P_{lac}	cm/min	-	-	5E-4 (5)	-
Surface Area	A	cm^2	-	-	1.12	-
Volume	V	ml	0.2	1.6	Apical: 0.5<br>Basal: 1.5	1
Systemic Flow Rate	Q_{sys}	ml/day	-	-	-	50
Flow Partitioning	Q	ml/day	$0.21*Q_{mix}$	$0.26*Q_{mix}$	$0.53*Q_{mix}$	-
Glucose consumption	k_{gluc}	1/day	0.09	0.02	0.4	-
Lactate production	k_{lac}	1/day	0.1	0.06	0.8	-

Metabolomics and Proteomics

Gut Intracellular Metabolomics (Basic Scan, CE & LC-TOFMS)

CE-TOFMS measurement was carried out using an Agilent CE Capillary Electrophoresis System equipped with an Agilent 6210 Time of Flight mass spectrometer, Agilent 1100 isocratic HPLC pump, Agilent G1603A CE-MS adapter kit, and Agilent G1607A CE-ESI-MS sprayer kit (Agilent Technologies, Waldbronn, Germany). The systems were controlled by Agilent G2201AA ChemStation software version B.03.01 for CE (Agilent Technologies, Waldbronn, Germany). The metabolites were analyzed by using a fused silica capillary (50 µm *i.d.* × 80 cm total length), with commercial electrophoresis buffer (Solution ID: H3301-1001 for cation analysis and H3302-1021 for anion analysis, Human Metabolome Technologies) as the electrolyte. The sample was injected at a pressure of 50 mbar for 10 sec (approximately 10 nL) in cation analysis and 25 sec (approximately 25 nL) in anion analysis. The spectrometer was scanned from *m/z* 50 to 1,000. Other conditions were as in the described previously (6-8).

LC-TOFMS measurement was carried out using an Agilent LC System (Agilent 1200 series RRLC system SL) equipped with an Agilent 6230 Time of Flight mass spectrometer (Agilent Technologies, Waldbronn, Germany). The systems were controlled by Agilent G2201AA ChemStation software version B.03.01 for CE (Agilent Technologies, Waldbronn, Germany). The cationic and anionic compounds were measured by using ODS column (2×50 mm, 2 µm) according to the methods described previously (9).

Peaks were extracted using automatic integration software MasterHands (Keio University, Tsuruoka, Japan) in order to obtain peak information including *m/z*, migration time for CE-TOFMS measurement (MT) or retention time for LC-TOFMS measurement (RT), and peak area (10). Signal peaks corresponding to isotopomers, adduct ions, and other product ions of known metabolites were excluded, and remaining peaks were annotated with putative metabolites from the HMT metabolite database based on their MTs/RTs and *m/z* values determined by TOFMS. The tolerance range for the peak annotation was configured at ±0.5 min for MT and ±10 ppm for *m/z*. In addition, peak areas were normalized against those of the internal standards and then the resultant relative area values were further normalized by sample amount.

Metabolomics Data Analysis

Hierarchical cluster analysis (HCA) and principal component analysis (PCA) were performed by HMT's proprietary software, PeakStat and SampleStat, respectively.

Detected metabolites were plotted on metabolic pathway maps using VANTED (Visualization and Analysis of Networks containing Experimental Data) software (11).

Gut Intracellular Proteomics

Tandem Mass Tag (TMT) Labeling

25 µg of each digested sample was added to 50 µL of 100 mM HEPES, pH 8.5 buffer. A reference pooled sample which was composed of equal amounts of material from all samples was also generated to link all 3 TMT experiments. Isobaric labeling of the samples was performed using 10-plex tandem mass tag (TMT) reagents (Thermo-Fisher Scientific, Rockford, IL). All individual and pooled samples were labeled according to the TMT 10plex reagent kit instructions. Briefly, TMT regents were brought to room temperature and dissolved in anhydrous acetonitrile. Peptides were labeled by the addition of each label to its respective digested sample. Labeling reactions were incubated with shaking for 1 h at room temperature. Reactions were terminated with the addition of hydroxylamine. Subsequent labeled digests were combined into a new 2 mL microfuge tube, acidified with formic acid, subjected to Sep-Pak C18 solid phase extraction and dried down.

High pH Reverse Phase Fractionation

The dried peptide mixture was dissolved in 110 µL of mobile phase A (10 mM ammonium formate, pH 9.0). 100 µL of each sample was injected onto a 2.1 x 150 mm XSelect CSH C18 column (Waters, Waltham, MA) equilibrated with 3% mobile phase B (10 mM ammonium formate, 90% ACN). Peptides were separated into 60 peptide fractions using a 47 min linear gradient from 3% to 50% B at a flow rate of 0.2 mL/min. The 60 fractions were pooled into 10 samples in which every 10th fraction (ie: 1, 11, 21, 31, 41, 51; six fractions total) was combined. The 10 pooled samples were acidified and dried down prior to LC-MS analysis.

LC-MS Analysis

Each fraction was resuspended in 10 µL 1% acetonitrile/1% formic acid. 5 µL was analyzed by LC-MS with a Dionex RSLCnano HPLC coupled to a Q-Exactive (Thermo-Fisher Scientific, San Jose, CA) mass spectrometer. Peptides were resolved using a 75 µm x 25 cm PepMap C18 column (Thermo-Fisher Scientific, San Jose, CA) with a 2-hour gradient from 3% solvent B

(acetonitrile with 0.1% formic acid) / 97% solvent A (0.1% formic acid) to 30% B / 70% A in 85 min at a flow rate of 260 nL. The instrument was operated in the data-dependent mode in which each MS1 scan was followed by high-collision-dissociation (HCD) of the 15 most intense precursor ions with dynamic exclusion for 30 s. MS1 resolution was set to 70,000 with an automatic gain control (AGC) target of 3E6, and a max injection time of 100 ms. For MS2 scans, the resolution was set to 35,000 with an automatic gain control (AGC) target of 1E5. Normalized collision energy was set to 30% with a max injection time of 100 ms. The isolation window for MS2 precursors was set to 2.0 Th. Precursors with singly or unassigned charge state were excluded from triggering MS2 events.

Data Analysis

All MS/MS data were analyzed using Proteome Discoverer 2.1 (Thermo-Fisher Scientific, San Jose, CA). The Sequest HT search engine in the Proteome Discover was set to search human database (Uniprot.org). The digestion enzyme was set as trypsin. The HCD MS/MS spectra were searched with a fragment ion mass tolerance of 0.02 Da and a parent ion tolerance of 10 ppm. Oxidation of methionine was specified as a variable modification, while carbamidomethyl of cysteine and TMT labeling were designated at lysine residues or peptide N-termini and specified in Proteome Discoverer as static modifications. MS/MS based peptide and protein identifications and quantification results were initially generated in Proteome Discover 2.1 and later uploaded to Scaffold (version Scaffold_4.8.2 Proteome Software Inc., Portland, OR) for final **TMT quantification** and data visualization.

REFERENCES

1. Chen WLK, Edington C, Suter E, Yu J, Velazquez JJ, Velazquez JG, et al. Integrated gut/liver microphysiological systems elucidates inflammatory inter-tissue crosstalk. Biotechnol Bioeng. 2017.

2. Domansky K, Inman W, Serdy J, Dash A, Lim MH, Griffith LG. Perfused multiwell plate for 3D liver tissue engineering. Lab Chip. 2010;10(1):51-8.

3. Mathur A, Loskill P, Shao K, Huebsch N, Hong S, Marcus SG, et al. Human iPSC-based cardiac microphysiological system for drug screening applications. Sci Rep. 2015;5:8883.

4. Sun D, Lennemas H, Welage LS, Barnett JL, Landowski CP, Foster D, et al. Comparison of human duodenum and Caco-2 gene expression profiles for 12,000 gene sequences tags and correlation with permeability of 26 drugs. Pharm Res. 2002;19(10):1400-16.

5. Lam WK, Felmlee MA, Morris ME. Monocarboxylate transporter-mediated transport of gamma-hydroxybutyric acid in human intestinal Caco-2 cells. Drug Metab Dispos. 2010;38(3):441-7.

6. Soga T, Heiger DN. Amino acid analysis by capillary electrophoresis electrospray ionization mass spectrometry. Anal Chem. 2000;72:1236-41.

7. Soga T, Ueno Y Fau - Naraoka H, Naraoka H Fau - Ohashi Y, Ohashi Y Fau - Tomita M, Tomita M Fau - Nishioka T, Nishioka T. Simultaneous determination of anionic intermediates for Bacillus subtilis metabolic pathways by capillary electrophoresis electrospray ionization mass spectrometry. Anal Chem. 2002;74:2233-9.

8. Soga T, Ohashi Y, Ueno Y, Naraoka H, Tomita M, Nishioka T. Quantitative Metabolome Analysis Using Capillary Electrophoresis Mass Spectrometry. Journal of Proteome Research. 2003;2(5):488-94.

9. Ooga T, Sato H Fau - Nagashima A, Nagashima A Fau - Sasaki K, Sasaki K Fau - Tomita M, Tomita M Fau - Soga T, Soga T Fau - Ohashi Y, et al. Metabolomic anatomy of an animal model revealing homeostatic imbalances in dyslipidaemia. Mol Biosyst. 2011;7:1217-23.

10. Sugimoto M Fau - Wong DT, Wong Dt Fau - Hirayama A, Hirayama A Fau - Soga T, Soga T Fau - Tomita M, Tomita M. Capillary electrophoresis mass spectrometry-based saliva metabolomics identified oral, breast and pancreatic cancer-specific profiles. Metabolomics. 2009;6(1):78-95.

11. Junker BH, Klukas C Fau - Schreiber F, Schreiber F. VANTED: a system for advanced data analysis and visualization in the context of biological networks. BMC Bioinformatics. 2006;7(1471-2105 (Electronic)):109.
